# TMEM11 regulates cardiomyocyte proliferation and cardiac repair via METTL1-mediated m^7^G methylation of ATF5 mRNA

**DOI:** 10.1038/s41418-023-01179-0

**Published:** 2023-06-07

**Authors:** Xin-Zhe Chen, Xin-Min Li, Shi-Jun Xu, Shen Hu, Tao Wang, Rui-Feng Li, Cui-Yun Liu, Jun-Qiang Xue, Lu-Yu Zhou, Yun-Hong Wang, Pei-Feng Li, Kun Wang

**Affiliations:** 1 Institute for Translational Medicine, The Affiliated Hospital of Qingdao University, College of Medicine, Qingdao University, Qingdao, 266021 China; 2 Department of Cardiac Surgery, Beijing Anzhen Hospital, Capital Medical University, 100029 Beijing, China; 3 Department of Neurosurgery, Beijing Hospital, National Center of Gerontology, 100730 Beijing, China; 4 Department of Rehabilitation Medicine, the Affiliated Hospital of Qingdao University, Qingdao, 266000 China; 5 State Key Laboratory of Cardiovascular Disease, Heart Failure center, Fuwai Hospital, National Center for Cardiovascular Diseases, Chinese Academy of Medical Sciences, Peking Union Medical College, 100037 Beijing, China

**Keywords:** Epigenetics, Cardiovascular diseases

## Abstract

The mitochondrial transmembrane (TMEM) protein family has several essential physiological functions. However, its roles in cardiomyocyte proliferation and cardiac regeneration remain unclear. Here, we detected that TMEM11 inhibits cardiomyocyte proliferation and cardiac regeneration in vitro. TMEM11 deletion enhanced cardiomyocyte proliferation and restored heart function after myocardial injury. In contrast, TMEM11-overexpression inhibited neonatal cardiomyocyte proliferation and regeneration in mouse hearts. TMEM11 directly interacted with METTL1 and enhanced m^7^G methylation of *Atf5* mRNA, thereby increasing ATF5 expression. A TMEM11-dependent increase in ATF5 promoted the transcription of *Inca1*, an inhibitor of cyclin-dependent kinase interacting with cyclin A1, which suppressed cardiomyocyte proliferation. Hence, our findings revealed that TMEM11-mediated m^7^G methylation is involved in the regulation of cardiomyocyte proliferation, and targeting the TMEM11-METTL1-ATF5-INCA1 axis may serve as a novel therapeutic strategy for promoting cardiac repair and regeneration.

## Introduction

Cardiovascular disease is the leading cause of death worldwide, and its occurrence is highly correlated with age. Heart failure is a global challenge primarily caused by myocardial cell death [1–4]. The prevalence of heart failure with preserved ejection fraction, such as hypertensive cardiomyopathy, is increasing and is a major cause of hospitalization for heart failure. Myocardial infarction (MI) leads to a massive loss of functional cardiac cells, which is the basic pathological process that induces heart failure. Previous experimental studies have identified complex signal transduction processes that regulate cardiomyocyte death; however, novel therapeutic interventions are still lacking. As a novel therapeutic avenue, MI can be treated by inducing the proliferation of existing adult cardiomyocytes (ACMs), which produces new cardiomyocytes. Mature cardiomyocytes can reenter the cell cycle through dedifferentiation, proliferation, and redifferentiation to form new cardiomyocytes [5, 6]. Several proteins, such as HIPPO, YAP1, PITX2 and MEIS1, play vital roles in controlling the proliferation of adult mammalian cardiomyocytes [7–11]. The HIPPO signaling pathway is an evolutionarily conserved signaling pathway that restricts development and reproduction mainly by inhibiting the activity of the transcriptional coactivator YAP [10, 12]. Moreover, non-coding RNAs, including miRNAs and lncRNAs, play crucial roles in regulating cardiomyocyte proliferation and cardiac regeneration [13–16]. Although substantial progress has been made in understanding the molecular mechanisms of adult mammalian cardiomyocytes proliferation and cardiac regeneration, an in-depth evaluation of these molecular networks is required.

Mitochondria are highly dynamic organelles involved in various physiological activities, such as cell cycle, development, and morphological change [17]. The mitochondria of cardiomyocytes produce most of their chemical energy in the form of adenosine triphosphate (ATP), which is necessary for cardiac contractility and robust heart function [18]. Although mitochondria play a central role in the onset and progression of heart failure (HF) and other cardiovascular diseases (CVD), there is currently no treatment for mitochondrial dysfunction [19]. The mitochondrial transmembrane protein (TMEM) family has several essential physiological functions, notably autophagy, apoptosis, participation in signal transduction pathways, and the formation of plasma membrane ion channels [20–22]. TMEMs also play critical roles in many pathological processes. For instance, TMEMs can function as tumor suppressors [23, 24], and some TMEMs with predictive endoplasmic reticulum localization can be used for kidney cancer classification [25]. Down-regulation of the *TMEM88* gene can inhibit myocardial cell differentiation and promote endothelial cell differentiation [26]. However, the functions of TMEMs in the heart remain largely unknown, and their biological functions in cardiovascular diseases require further exploration.

Transcriptional and post-transcriptional regulation, including methylation, demethylation, and RNA splicing, have significant impact on mRNA turnover, which regulates protein expression diversity during cardiomyocyte proliferation and heart regeneration [27–33]. The N^7^-methylguanosine (m^7^G) modification present in the mRNA 5′ cap plays a fundamental role in the regulation of transcription elongation [34], mRNA splicing [35], polyadenylation [36], and translation [37], and therefore it is implicated in nearly all aspects of the mRNA life cycle. m^7^G is also present in tRNAs and rRNAs, where it is associated with human diseases [38, 39]. A recent study showed that methyltransferase METTL1 mediates m^7^G modification within mammalian mRNAs, which affects their translation [40]. Additionally, METTL1-dependent N7-methylation of guanosine regulates miRNA structure, biogenesis, and cell migration [41]. These findings indicate that RNA m^7^G modification is strongly associated with the gene regulatory networks involved in both physiological and pathological processes. However, the cellular and molecular mechanisms underlying dysregulation of m^7^G modifications in the heart are unknown.

Here, we investigated the previously unrecognized function of TMEM11 in regulating cardiomyocyte proliferation in the postnatal heart and uncovered its underlying mechanism. We demonstrated that TMEM11 suppressed cardiomyocyte cell cycle activity by directly interacting with METTL1 and increasing its RNA m^7^G-methylation activity, thereby upregulating activating transcription factor 5 (ATF5) expression through hypermethylation of *Atf5* mRNA. Further, upregulated ATF5 enhanced the expression of INCA1, an inhibitor of cyclin-dependent kinase (CDK) interacting with cyclin A1, which suppressed cardiomyocyte proliferation. Collectively, our study uncovered a novel mechanism by which TMEM11-dependent m7G modification regulates cardiomyocyte proliferation. Targeting the TMEM11-METTL1-ATF5 axis may be an effective strategy for improving heart regeneration after cardiac injury.

## Methods

### Primary cardiomyocytes culture and treatment

Cardiomyocytes were isolated from neonatal mice (1–2 days old), and we washed the newborn mouse with 75% alcohol. The hearts were surgically cut out and placed in a petri dish with pre-cooled PBS in a sterile environment. We cleaned the hearts with PBS three times, and we shredded the heart tissue with surgical scissors. The heart tissue was transferred to 10 ml of digestive juice (1.2 mg/ ml trypsin and 0.14 mg/ml collagenase II) and gently stirred in a 37 °C water bath for 6 min. After each digestion, we transferred the supernatant to a centrifuge tube containing serum, which was stored on ice, and we added new digestive juice again until the heart tissue disappeared. Centrifuge at 4 °C at 1000 rpm/min for 10 min, the supernatant was taken and re-suspended with F12/DMEM containing 10% serum. Centrifuge at 4 °C at 1000 rpm/min for 10 min, the supernatant was taken and filtered by 70 um filter. The filtered cardiomyocytes were placed in a 10 cm petri dish. Then the suspended cells were placed in a 5% CO_2_ humidified incubator at 37 °C for 1.5 h. Fibroblasts were attached to the wall, and the culture medium containing cardiomyocytes was collected into a new centrifuge tube. The supernatant was discarded in a 4 °C centrifuge at 1000 rpm/min for 10 minutes. And added 0.1 mm bromodeoxyuridine. According to the experimental design, the plates were placed in a humidified incubator with 5% CO_2_ at 37 °C. The next day, the culture medium in the petri dish was replaced, and we continued the culture for another day. On the third day, the cardiomyocytes transfected with adenovirus for 6 h were cultured in a new medium for 24 h.

### Generation of TMEM11 knockout (TMEM11 KO) mice and TMEM11 transgenic (TMEM11 Tg) mice

All animal experiments were approved by the Institutional Review Board (IRB) of Qingdao University, and the procedures followed were by the guidelines of Qingdao University institutions. TMEM11 knockout mice were generated using the CRISPR/Cas9 technology to modify Tmem11 gene from Nanjing GemPharmatech company in China. The Tmem11 gene has 3 transcripts. According to the structure of Tmem11 gene, exon2 of Tmem11-201 transcript is recommended as the knockout region. The region contains most of the coding sequence. Knock out the region will result in disruption of protein function. The brief process is as follows: sgRNA was transcribed in vitro. Cas9 and sgRNA were microinjected into the fertilized eggs of C57BL/6JGpt mice. Fertilized eggs were transplanted to obtain positive F0 mice which were confirmed by PCR and sequencing. A stable F1 generation mouse model was obtained by mating positive F0 generation mice with C57BL/6JGpt mice. The following primer sequences were used to amplify the genomic DNA fragments containing the CRISPR/Cas9 target sites and TMEM11 knockout. The forward primer(F1) was 5′-CTCCAATCTGAATACATCCAAAGTCC-3′; the reverse primer(R1) was 5′-GGCATTTCAGAATCTGGGTTAGTACAC-3′; the forward primer(F2) was 5′-TGCTGTCTTACCTGTGAACTTGAGAGA-3′; the reverse primer(R2) is 5′-CTGTTTGTCAGGAGAAATGAGTGAGC-3′.

TMEM11 transgenic mice were generated using the microinjection technology from Nanjing GemPharmatech company in China. The brief process is as follows: In order to construct heart-specific TMEM11 overexpression transgenic mice (TMEM11 Tg), the mouse TMEM11 gene fragment was cloned into a vector (pαMHC-clone26) under the control of the α-myosin heavy chain (MHC) promoter. The constructed transgenic vector was microinjected into the fertilized eggs of C57BL/6JGpt mice. Microinjection was performed according to the standard protocols. Fertilized eggs were transplanted to obtain positive F0 mice which were confirmed by PCR and sequencing. A stable F1 generation mouse model was obtained by mating positive F0 generation mice with C57BL/6JGpt mice. The forward primer(F1) was 5′-GGAAGGAGGCGTCTGGGTC-3′; the reverse primer(R1) was 5′-TCATACTCCACCTGGTACTTGCAG-3′; the forward primer(F2) was 5′-CTAGGCCACAGAATTGAAAGATCT-3′; the reverse primer(R2) is 5′-CTAGGCCACAGAATTGAAAGAT CT-3′.

### MeRIP-qPCR

Total RNA was extracted from cardiomyocytes or tissues. RNase MiniElute Kit segmented RNA, we added 50 ul protein A/G magnetic beads to the segmented RNA sample, we added 500 ul 1x IP buffer, we used the pipette to blow and mix, and magnetic beads were suspended again. Place the Eppendorf tube on the magnetic rack until the liquid is clear and discard the supernatant. We added 200 ul 1 x IP buffer to re-suspend protein A/G magnetic beads. Rotate and incubate at room temperature for 30 minutes. Place the Eppendorf tube on the magnetic rack until the liquid is clear and discard the supernatant. Add MeRIP reaction mixture, gently pipette up and down several times, make protein A/G magnetic beads completely re-suspended. Rotate and incubate at 4 °C for 2 hours. Place the Eppendorf tube on the magnetic rack until the liquid is clear and discard the supernatant. Add 100 ul of eluent into the Eppendorf tube, gently pipette up and down several times to make the magnetic beads wholly suspended. Under the condition of 4 °C, continuously shake and incubate for 1 h. Place the Eppendorf tube on a magnetic stand until the liquid is clear. The supernatant containing elution RNA fragments was transferred to a new 1.5 mL Eppendorf tube. After elution, the RNA sample was purified by RNase MiniElute Kit, the RNA sample after MeRIP was obtained. Reverse transcription was performed on the obtained samples, and qPCR was used to detect the expression.

### Immunofluorescence

The processed cardiomyocytes or heart tissue sections were fixed with 4% paraformaldehyde for 15 min, washed with PBS three times, and treated with 0.1% Triton X-100 for 15 min, washed with PBS three times, and incubated with 5% BSA solution for 30 min. Specific antibody binding protein signal was used, and the antibodies were as follows: Anti-Ki67 (abcam, Cat: AB16667, 1:200); Anti-Aurora B (abcam, Cat: AB2254, 1:200); Anti-histone H3 (abcam Cat: AB5176, 1:200); Anti-cardiac Troponin T (abcam, Cat: AB8295, 1:200); TMEM11 Rabbit pAb, (Abclonal, A17578, 1:100); Anti-LC3B (abcam,Cat:ab192890,1:1000); Caspase-3 (D3R6Y) Rabbit mAb, (cell signaling technology,Cat:#14220,1:1000). The fluorescence signal was detected by a Leica inverted two-photon laser confocal scanning microscope.

### MeRIP-sequencing

RNA was extracted from WT and TMEM11 Tg mice hearts and quantified (using NanoDrop ND-1000). M^7^G RNA-Seq service was provided by Cloudseq Biotech Inc. (Shanghai, China). Briefly, total RNA was decapped with mRNA decapping enzyme (New England Biolabs), and then subjected to m^7^G RNA immunoprecipitation with the GenSeqTM m^7^G RNA IP Kit (GenSeq Inc., China) by following the manufacturer’s instructions. Both the input sample without immunoprecipitation and the m^7^G IP samples were used for RNA-seq library generation with NEBNext® Ultra II Directional RNA Library Prep Kit (New England Biolabs, Inc., USA). The library quality was evaluated with BioAnalyzer 2100 system (Agilent Technologies, Inc., USA). Library sequencing was performed on an illumina Hiseq instrument with 150 bp paired-end reads. Paired-end reads were harvested from Illumina HiSeq 4000 sequencer, and were quality controlled by Q30. After 3′ adaptor-trimming and low quality reads removing by cutadapt software (v1.9.3). First, clean reads of all libraries were aligned to the reference genome (UCSC MM10) by Hisat2 software (v2.0.4). Methylated sites on RNAs (peaks) were identified by MACS software. Differentially methylated sites were identified by diffReps. These peaks identified by both softwares overlapping with exons of mRNA were figured out and choosed by home-made scripts. GO and Pathway enrichment analysis were performed by the differentially methylated protein coding genes.

### Statistical analysis

Data were expressed as the mean ± SD. Statistical analysis was performed using GraphPad Prism 7.0 (GraphPad Software Inc., San Diego, CA). Student’s t-test was used for the comparison of statistical significance between two groups and all tests were determined by unpaired two-sided tests. Comparisons between multiple groups were assessed by one-way ANOVA with Tukey’s multiple comparisons test or two-way ANOVA with Bonferroni’s multiple comparisons test. The experiments were repeated independently at least three times with similar results and *p* < 0.05 was considered statistically significant.

## Results

### Inhibition of TMEM11 promotes cardiomyocyte proliferation in vitro

**Fig. 1:**
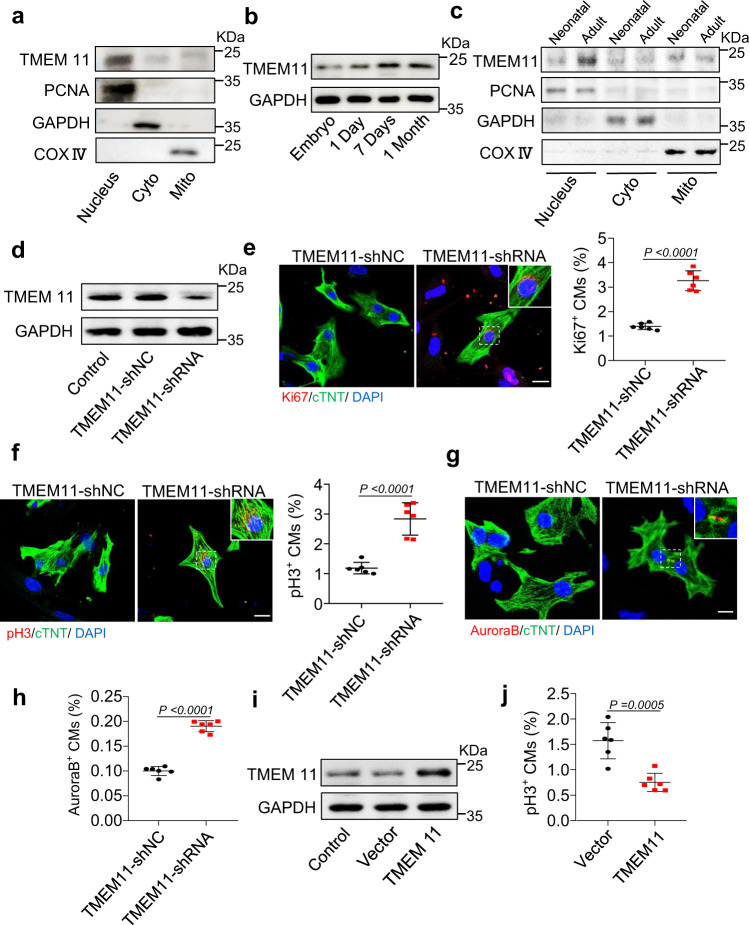
TMEM11 participates in the regulation of proliferation in cardiomyocytes. **a** Representative western blots showing the expression of TMEM11 in the nucleus, cytoplasm and mitochondria of cardiomyocytes (*n* = 3 independent experiments). **b** Representative western blots showing the expression of TMEM11 in hearts at different ages of mice (*n* = 4 mice). **c** Representative western blots showing the expression of TMEM11 in the nucleus, cytoplasm and mitochondria of cardiomyocytes (*n* = 3 mice). **d**–**h** Neonatal mice cardiomyocytes were infected with adenovirus harboring TMEM11-shRNA or its control (TMEM11-shNC) for 48 h. **d** The expression levels of TMEM11 mRNA assessed by immunoblot (*n* = 3 independent experiments). **e** Representative confocal images of Ki67-positive cardiomyocytes (Bar = 20 μm) and quantification of Ki67-positive (right) cells (*n* = 6 independent experiments). **f** Representative confocal images of pH3-positive cardiomyocytes and quantification of pH3-positive cells in TMEM11-shNC and TMEM11-shRNA infected cardiomyocytes (Bar = 20 μm) (*n* = 6 independent experiments). Representative confocal images of Aurora-B-positive cardiomyocytes (**g**) and quantification (**h**) of Aurora-B-positive cells in TMEM11-shNC and TMEM11-shRNA infected cardiomyocytes (Bar = 20 μm) (*n* = 6 independent experiments). **i**, **j** Neonatal mice cardiomyocytes were infected with adenovirus harboring negative control (Vector) or mouse TMEM11 gene (TMEM11) for 48 h. **i** Representative western blots showing the expression of TMEM11 (*n* = 4 independent experiment). **j** PH3-positive cardiomyocytes were measured in Vector and TMEM11 treated cardiomyocytes (*n* = 6 independent experiment). Data are presented as the mean ± s.d. Two-sided Student’s *t* test (**e**, **f**, **h** and **j**) was performed.

To investigate the potential role of mitochondrial proteins in regulating cardiomyocyte proliferation, we reviewed the mitochondrial proteins previously reported in the literature and analyzed their location and expression in the heart. Interestingly, we observed that the mitochondrial inner membrane protein TMEM11 was distributed in the nucleus, cytoplasm, and mitochondria of cardiomyocytes **(**Fig. 1a and Supplementary Fig. 1a). The expression pattern of the TMEM11 protein in the postnatal hearts of mice revealed that TMEM11 protein levels were relatively low at birth and increased during adulthood (Fig. 1b), indicating that TMEM11 expression might be associated with the loss of cardiac regenerative capacity in adult mice. Furthermore, increased expression of TMEM11 was mainly observed in the nucleus, whereas no significant increase was detected in the cytoplasm or mitochondria in the adult mouse heart (Fig. 1c). Also, TMEM11 was abundantly expressed in the heart (Supplementary Fig. 1b) and was significantly higher in cardiomyocytes than in fibroblasts (Supplementary Fig. 1c). Thus, we speculated that TMEM11 has a potential regulatory role in cardiomyocyte proliferation and cardiac regeneration.

To verify the role of TMEM11 in regulating cardiomyocyte proliferation, we constructed a recombinant adenoviral vector harboring *a Tmem11*-targeting shRNA (TMEM11-shRNA) to silence TMEM11 expression in neonatal mouse cardiomyocytes (Fig. 1d). Silencing of TMEM11 increased the expression of proliferation markers in cardiomyocytes, as indicated by an increase in Ki67-positive (Fig. 1e), mitosis marker phospho-histone H3 (pH3)-positive (Fig. 1f), and cytokinesis marker Aurora B-positive cells (Fig. 1g, h). Next, we overexpressed TMEM11 in cardiomyocytes (Fig. 1i), and exogenous TMEM11 expression significantly reduced the expression of proliferation markers (Fig. 1j, Supplementary Fig. 1d–f). In addition, we also assessed whether TMEM11 affects mitochondrial functions. Our results demonstrated that knockdown of TMEM11 in cardiomyocytes did not change the mitochondrial membrane potential (Supplementary Fig. 2a, b), which is an important indicator of mitochondrial activity, and TMEM11 had no effect on apoptosis or autophagy (Supplementary Fig. 2c, d). Together, these data suggest that ablation of TMEM11 promotes cardiomyocyte proliferation, whereas its overexpression inhibits cell cycle progression.

### TMEM11 deficiency reactivates cardiomyocytes proliferation in the adult hearts

**Fig. 2:**
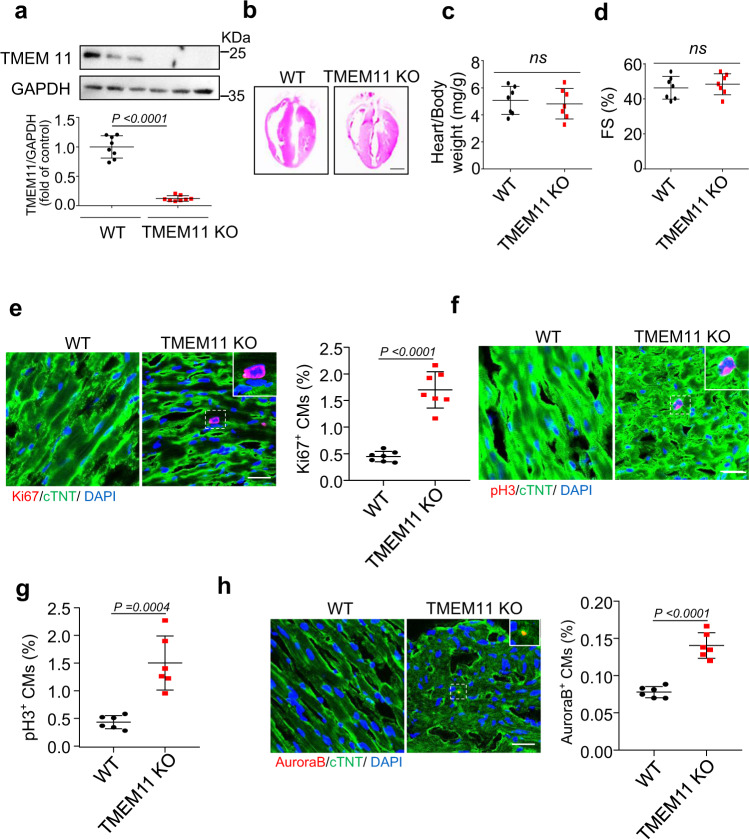
TMEM11 mediates cardiomyocytes proliferation in adult hearts. **a** Western blot image (top) and statistical data (bottom) showing the myocardial level of TMEM11 in wild type (WT) and TMEM11 KO adult mice hearts (*n* = 8 mice). **b–h** TMEM11 silencing increases cardiomyocytes proliferation in adult mice hearts. Representative images of gross morphology and haematoxylin-eosin stained transverse sections of hearts from WT and TMEM11 KO mice (Bar = 2 mm) (**b**). Heart to body weight ratio (**c**) and echocardiography analysis of fractional shortening (FS) (**d**) in WT and TMEM11 KO mice (*n* = 7 mice). **e** Representative confocal images of Ki67-positive cardiomyocytes (red, Bar = 25 μm) and quantification of Ki67-positive (right) cells (*n* = 7 mice per group). cTNT (green) mark cardiomyocytes and DAPI (blue) labels nuclei. **f**, **g** Representative confocal images and qualification of pH3-positive cardiomyocytes (red, Bar = 25 μm) in WT and TMEM11 KO hearts. **h** Aurora-B-positive (red) cardiomyocytes was calculated in WT and TMEM11 KO hearts. cTNT marks cardiomyocytes and DAPI labels nuclei (Bar = 25 μm). (*n* = 6 mice per group). Data are presented as the mean ± s.d. Two-sided Student’s *t* test (**a**, **c**–**e**, **g** and **h**) was performed.

To investigate the effects of TMEM11 knockdown on cardiomyocyte proliferation in vivo, we generated TMEM11 knockout (TMEM11 KO) mice, and the deletion of TMEM11 in mice hearts was confirmed by western blot analysis (Fig. 2a). Cardiac tissue morphology, heart-to-body weight ratio, and heart function were not significantly different between WT and TMEM11 KO mice under physiological conditions (Fig. 2b–d, and Supplementary Fig. 3a, b). We analyzed the cross-sectional area and number of cardiomyocytes in the TMEM11 KO and WT mice. Cardiomyocytes in the TMEM11 KO hearts were smaller in size (Supplementary Fig. 3c, d), and the number of cardiomyocytes was increased in the TMEM11 KO hearts (Supplementary Fig. 3e). In addition, no hypertrophic effects were observed in TMEM11 KO mouse hearts (Supplementary Fig. 3f, g). Further, knockout of TMEM11 increased the number of Ki67-positive cells compared to those in WT mice (Fig. 2e). Likewise, the numbers of pH3-positive (Fig. 2f, g) and Aurora B-positive cells (Fig. 2h) were remarkably higher in *Tmem11*-deleted adult hearts than in WT mice. Together, these results indicated that the loss of TMEM11 enhanced cardiomyocyte proliferation in vivo.

### Knockout of TMEM11 promotes cardiomyocyte proliferation and cardiac regeneration after ischemic injury

**Fig. 3:**
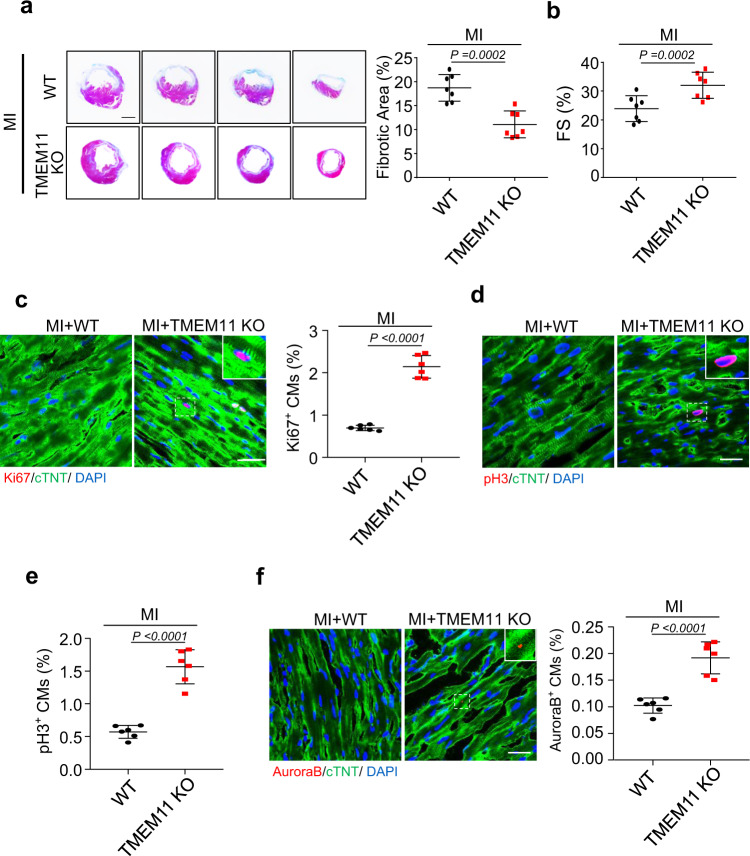
TMEM11 deletion promotes cardiomyocytes proliferation and improves cardiac function after MI injury. **a**–**f** 10-weeks old wild type (WT) and TMEM11 KO mice were subjected to MI and heart samples were collected at 8 weeks post-MI. **a** Representative images of Masson’s trichrome-stained cross-sections of hearts (Bar = 2 mm) (left panel). The analysis of the percentage of the fibrotic area after MI injury (right panel, *n* = 7 mice per group). **b** Echocardiography analysis of left ventricular function (fractional shortening, FS%) after MI (*n* = 7 mice). **c** Representative confocal images of Ki67-positive cardiomyocytes (red, Bar = 25 μm) and quantification of Ki67-positive (right) cells (*n* = 6 mice per group). cTNT (green) marks cardiomyocytes and DAPI (blue) labels nuclei. **d**, **e** Representative confocal images and qualification of pH3-positive cardiomyocytes (red, Bar = 25 μm) in WT and TMEM11 KO hearts (*n* = 6 mice per group). **f** Aurora-B-positive (red) cardiomyocytes was calculated in WT and TMEM11 KO hearts. cTNT marks cardiomyocytes and DAPI labels nuclei (Bar = 25 μm). (*n* = 6 mice per group). Data are presented as the mean ± s.d. Two-sided Student’s *t* test (**a**–**c**, **e** and **f**) was performed.

To further validate the impact of TMEM11 silencing on cardiac regeneration in the adult heart, TMEM11 KO and WT mice were subjected to MI induced by coronary artery ligation (Supplementary Fig. 4a). TMEM11 KO significantly reduced scar size compared to that in WT hearts (Fig. 3a), and the left ventricle function was improved in TMEM11 KO mice after MI (Fig. 3b, Supplementary Fig. 4b, c), indicating that TMEM11 silencing mediates the reparative potential of cardiomyocytes after cardiac injury. Meanwhile, the numbers of Ki67-positive, pH3-positive, and Aurora B-positive cells were higher in TMEM11 KO hearts after MI injury (Fig. 3c–f), which suggest that depletion of TMEM11 improved myocardial repair and cardiac function after MI injury by inducing cardiomyocyte proliferation. Furthermore, increased TMEM11 expression (Supplementary Fig. 4d) was observed upon MI injury, and there was no significant change in TMEM11 levels following TAC treatment (Supplementary Fig. 4e). Collectively, these data confirm that suppression of TMEM11 promoted cardiac regeneration after ischemic injury in mice.

### TMEM11 overexpression inhibits cardiomyocyte proliferation and cardiac regeneration in neonatal hearts

**Fig. 4:**
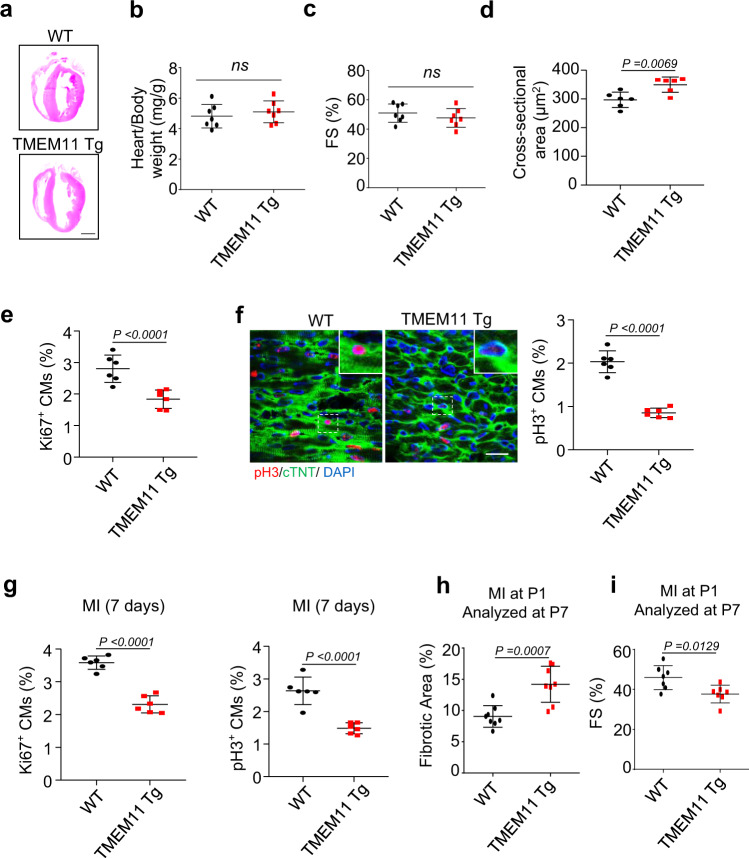
TMEM11 overexpression inhibits cardiomyocytes proliferation and cardiac regeneration in neonatal hearts. **a** Representative images of haematoxylin-eosin stained transverse sections of WT and TMEM11 transgenic (TMEM11 Tg) adult mice hearts (Bar = 2 mm). **b** Heart to body weight ratio in WT and TMEM11 Tg adult mice hearts (*n* = 7 mice per group). **c** The left ventricular fractional shortening (FS%) in WT and TMEM11 Tg adult mice hearts (*n* = 7 mice per group). **d** Cardiomyocytes surface area was measured in WT and TMEM11 Tg adult mice hearts (*n* = 6 mice per group). **e** Quantification of the number of Ki67-positive cardiomyocytes in WT and TMEM11 Tg heart sections at 7 days after birth (*n* = 6 mice per group). **f** Representative confocal images and qualification of pH3-positive cardiomyocytes (red, Bar = 25 μm) in WT and TMEM11 Tg heart sections at 7 days after birth (*n* = 6 mice per group). (**g–i**) WT and TMEM11 Tg neonatal mice (P1) were subjected to MI. **g** Quantification of pH3-positive and Ki67-positive cardiomyocytes in heart sections at 7 days after MI (*n* = 6 mice per group). **h** Analysis of the percentage of the fibrotic area (*n* = 8 mice per group). **i** Echocardiography analysis of fractional shortening (FS, *n* = 7 mice per group) at P7 post-MI. Data are presented as the mean ± s.d. Two-sided Student’s *t* test (**b**–**i**) was performed.

We next explored whether TMEM11 overexpression suppressed cardiomyocyte proliferation and cardiac regeneration in neonatal hearts. We generated cardiac-specific TMEM11 transgenic (TMEM11 Tg) mice (Supplementary Fig. 5a). Under physiological conditions, there were no differences in cardiac morphology, heart-to-body weight ratio, or hypertrophic marker expression and cardiac function between the Tg and WT mice in the adult mice hearts (Fig. 4a–c, Supplementary Fig. 5b–d). However, cardiomyocyte size increased in the hearts of Tg mice compared to that in WT mice (Fig. 4d). Besides, the number of Ki67-positive (Fig. 4e) or pH3-positive cells (Fig. 4f) were significantly reduced in the hearts of 7-day old Tg mice compared with that in WT mice, indicating that TMEM11 overexpression inhibits cardiomyocyte proliferation in mice. To further investigate the effect of TMEM11 overexpression on the regenerative capacity of neonatal hearts, 1-day old TMEM11 Tg mice were subjected to MI, and analyses were carried out on day 7 (P7). The number of Ki67-positive and pH3-positive cells (Fig. 4g, Supplementary Fig. 5e, f) decreased while the fibrotic area increased in TMEM11 Tg mice compared with that in WT mice (Fig. 4h). In addition, the cardiac function in TMEM11 Tg mice was impaired compared to that of WT controls following MI injury (Fig. 4i, Supplementary Fig. 5g). Together, these findings indicate that TMEM11 overexpression hindered cardiac regeneration by inhibiting cardiomyocyte proliferation in postnatal hearts.

### TMEM11 interacts with METTL1 and regulates its RNA m^7^G-methylation activity

**Fig. 5:**
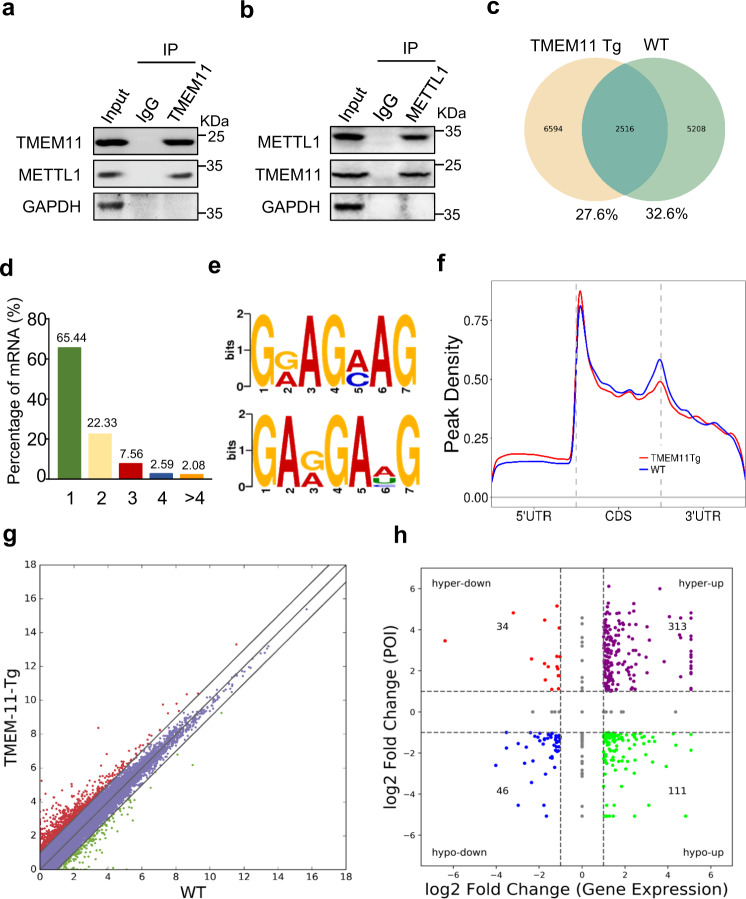
Transcriptome-wide m^7^G methylome analyses (using m^7^G-Seq) in TMEM11 Tg mice heart. Immunoprecipitation assessment using anti-TMEM11 antibody (**a**) and anti- METTL1 antibody (**b**) showing the interaction of TMEM11 and METTL1. Representative immunoblots from four independent experiments with similar results. **c**–**f** m^7^G methylated RNA immunoprecipitation and sequencing (MeRIP-seq) was performed in TMEM11 Tg and WT mice hearts. **c** Numbers of m^7^G peaks detected in TMEM11 Tg (left circle) and WT (right circle) mice hearts. **d** Percentage of mRNAs with different numbers of m^7^G peaks. **e** Sequence motifs enriched within m^7^G peaks identified by MeRIP-seq in TMEM11 Tg mice hearts. **f** Metagene profile showing the distribution of m^7^G peaks across the length of transcripts composed of three rescaled non-overlapping segments 5′UTR, CDS and 3′UTR in TMEM11 Tg and WT mice hearts. **g** Scatter plot of differential expression of mRNAs assessed from RNA-seq data. Red dots denote up-regulated genes and green dots denote down-regulated genes. **h** Correlation between the level of gene expression and changes in m^7^G level in TMEM11 Tg mice hearts compared to WT.

It was our first priority to investigate the proteins that interact with TMEM11 to better understand how TMEM11 regulates cardiomyocyte proliferation. Our experiments involved making protein immunoprecipitations from isolated cardiomyocytes and performing liquid chromatography tandem mass spectrometry (LC-MS/MS) on the samples (Supplementary Table 1). Among the proteins enriched with the anti-TMEM11 antibody, we identified METTL1, an RNA methyltransferase that specifically methylates N7-guanosine (Supplementary Fig. 6a). We further studied METTL1, considering that the function of m^7^G mRNA methylation in cardiomyocyte proliferation and cardiac regeneration remains unknown. We performed immunoprecipitation followed by western blotting and confirmed the direct interaction of TMEM11 and METTL1 in vivo (Fig. 5a, b). To determine whether TMEM11 regulates METTL1-dependent m^7^G-methylation in cardiomyocytes, we performed m^7^G-methylated RNA immunoprecipitation sequencing (MeRIP-seq) in WT and TMEM11 Tg mouse hearts (Fig. 5c and Supplementary Table 2).

Among all the detected m^7^G mRNA transcripts, more than half (65.44%) contained one m^7^G peak, and 4.67% contained four or more m^7^G peaks (Fig. 5d). Sequence motif analysis revealed that m^7^G peaks were highly enriched in two conserved consensus motif sequences of METTL1 (Fig. 5e). In both WT and TMEM11 Tg mice, m^7^G peaks were predominantly distributed in coding sequences (CDSs), particularly near the start and stop codons. However, m^7^G peaks had relatively lower density in the area between CDS and 3’ UTR in TMEM11 Tg mice compared to those in WT mice (Fig. 5f and Supplementary Fig. 6b). Gene Ontology analysis showed that genes with upregulated m^7^G modifications were mainly involved in cellular metabolism, protein modification, and regulation of signal transduction (Supplementary Fig. 6c). Genes with downregulated m^7^G modifications were mainly involved in the negative regulation of cellular, biosynthetic, and developmental processes (Supplementary Fig. 6d). Next, we performed RNA-seq analysis in TMEM11 Tg and WT mouse hearts to explore the relationship between m^7^G modifications and gene expression (Fig. 5g) and plotted m^7^G peak data against RNA-seq gene expression data to correlate the gene expression level with the m^7^G modification level (Fig. 5h and Supplementary Table 3). We termed the upregulated peaks as hypermethylated m^7^G peaks, including hyper-down and hyper-up genes. We termed the downregulated peaks as hypomethylated m^7^G peaks, including hypo-down and hypo-up genes.

### TMEM11 promotes METTL1-dependent m^7^G modification of *Atf5* mRNA and enhances its expression

**Fig. 6:**
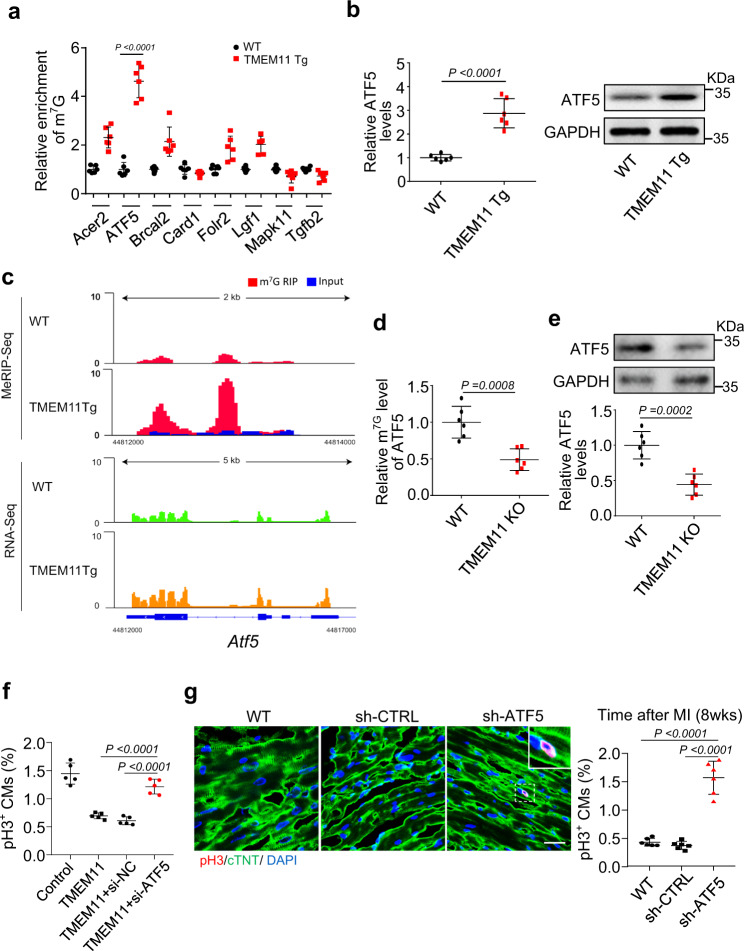
TMEM11 mediates m^7^G methylation of *Atf5* and its expression. **a** MeRIP-qPCR validation of m^7^G modification levels of genes which selected from the results of MeRIP-seq and mRNA-seq data in TMEM11 Tg and WT mice hearts (*n* = 6 mice per group). **b** Expression levels of *Atf5* mRNA and protein in WT and TMEM11 Tg mice hearts (*n* = 6 mice per group). **c** Integrative Genomics Viewer (IGV) tracks showing MeRIP-seq (upper panel) and mRNA-seq (bottom panel) read distribution in *Atf5* mRNA from TMEM11 Tg and WT mice hearts. **d** MeRIP-qPCR analysis in TMEM11 KO and WT mice hearts shows the m^7^G modification level in *Atf5* mRNA (*n* = 6 mice per group). **e** Expression levels of ATF5 protein (upper panel) and mRNA (lower panel) in TMEM11 KO and WT mice hearts (*n* = 6 mice per group). **f** Neonatal cardiomyocytes were infected with adenovirus harboring TMEM11, and transfected with si-NC or si-ATF5 for 48 h. Qualification of pH3-positive cardiomyocytes was calculated (*n* = 5 independent experiments). **g** Knockdown of ATF5 promotes cardiomyocyte proliferation following MI injury. AAV9 loaded with ATF5 shRNA (sh-ATF5) or its control (sh-CTRL) was administered to adult WT mice and subjected to MI, and heart samples were collected at 8 weeks post-MI. Representative confocal images and qualification of pH3-positive cardiomyocytes in heart sections at 8 weeks post-MI (Bar = 25 μm) (*n* = 6 mice per group). Data are presented as the mean ± s.d. One-way ANOVA (**f** and **g**) or two-sided Student’s *t* test (**a**, **b**, **d** and **e**) was performed.

We further investigated the potential downstream targets of METTL1 during cardiomyocyte proliferation in TMEM11 Tg hearts based on the results shown in Fig. 5h. We performed MeRIP-qPCR assays for the differentially methylated genes reported to be associated with proliferation. Our results revealed that the m^7^G modification of *Atf5* mRNA increased most significantly in TMEM11 Tg mice compared to WT mice (Fig. 6a). In addition, the expression of ATF5 significantly increased in TMEM11 Tg hearts (Fig. 6b). Integrative genomics viewer analysis of *Atf5* mRNA revealed a remarkable increase in m^7^G peaks, along with increased expression of *Atf5* mRNA, in TMEM11 Tg hearts (Fig. 6c). In contrast, m^7^G modification in *Atf5* mRNA decreased (Fig. 6d), along with decreased expression of *Atf5* mRNA and protein, in TMEM11 KO hearts (Fig. 6e). These results prompted us to select ATF5 as a potential target of the TMEM11-METTL1 axis in the regulation of cardiomyocyte proliferation.

We examined whether ATF5 acts as a downstream target of TMEM11 by regulating the proliferation of neonatal cardiomyocytes. TMEM11 overexpression inhibited cardiomyocyte proliferation, which was rescued by transfection with siRNA-ATF5 (Supplementary Figs. 7a, b and 6f). In addition, the knockdown of ATF5 using AAV9 system (sh-ATF5) in adult mice hearts (Supplementary Fig. 7c) promoted cardiomyocytes proliferation, improved cardiac function and reduced infarct size in MI-injured hearts (Fig. 6g and Supplementary Fig. 7d, e), indicating that ATF5 is involved in the regulation of cardiomyocyte proliferation and cardiac regeneration.

We investigated the mechanism by which TMEM11 regulates the expression of ATF5. We found that ATF5 expression was increased in MI-injured hearts (Supplementary Fig. 7f), but did not change significantly after TAC treatment (Supplementary Fig. 7g). The binding of METTL1 to *Atf5* mRNA was remarkably decreased in TMEM11 KO hearts compared to WT mice hearts (Supplementary Fig. 7h). Moreover, METTL1 overexpression increased the m^7^G modification of *Atf5* mRNA and elevated ATF5 expression, and these effects were reinforced by concomitant TMEM11 overexpression (Supplementary Fig. 7i). Furthermore, METTL1 knockdown attenuated the m^7^G modification of *Atf5* mRNA and its expression upon TMEM11 overexpression in vivo and in vitro (Supplementary Fig. 8a–c). Together, these results revealed that TMEM11 mediates m^7^G modification in *Atf5* mRNA through METTL1, and that this methylation enhances mRNA stability and translation capacity. As “YAP” is an important regulator of cardiac regeneration, we determine if YAP played a role in the TMEM11-mediated proliferation of cardiomyocytes. In our study, we found that TMEM11 did not affect the expression levels of YAP in cardiomyocytes. (Supplementary Fig. 8d, e). In addition, the interaction between TMEM11 and YAP was not detected by co-immunoprecipitation in cardiomyocytes (Supplementary Fig. 8f). In the results, it was found that YAP is not involved in the pathway of TMEM11-mediated cardiomyocyte proliferation.

### TMEM11 regulates INCA1 expression during cardiomyocyte proliferation

**Fig. 7:**
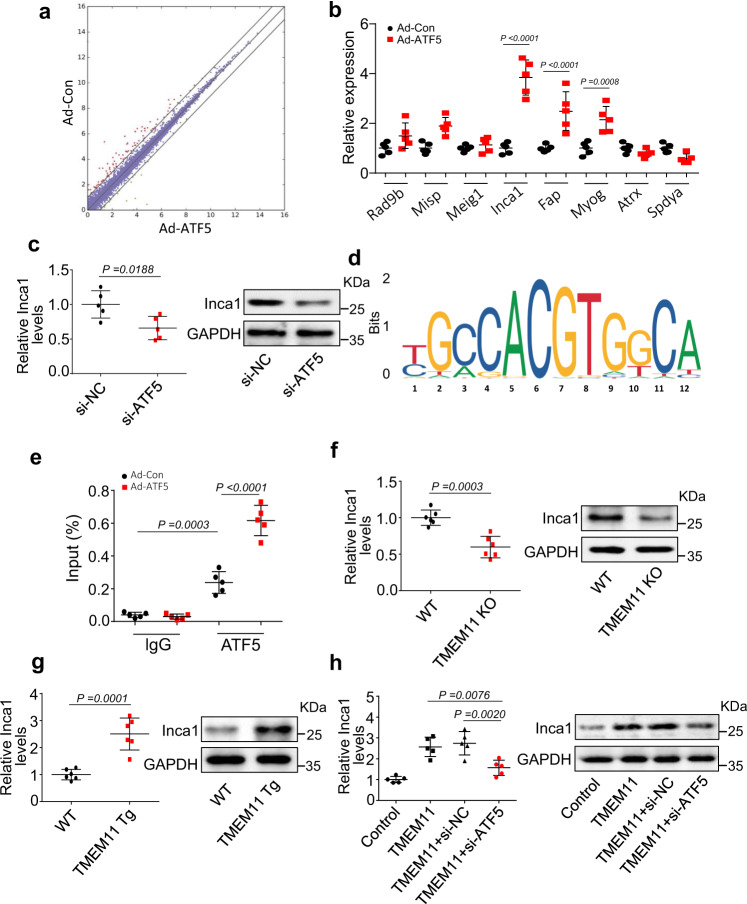
ATF5 regulates INCA1 expression during cardiomyocyte proliferation. **a** mRNA-seq analysis was performed in cardiomyocytes infected with or without adenovirus harboring ATF5 (Ad-ATF5 or Ad-Con). **b** qPCR validation of the expression levels of genes which were selected from the results of mRNA-seq data in Ad-ATF5 or Ad-Con treated cardiomyocytes (*n* = 5 independent experiments). **c** Cardiomyocytes were transfected with ATF5-siRNA (si-ATF5) and its control (si-NC). Inca1 mRNA and protein levels were evaluated by qRT-PCR and western blot (*n* = 5 independent experiments). **d** Motif analysis of the ATF5 bound regions. **e** Isolated neonatal cardiomyocytes were infected with adenovirus harboring ATF5 (Ad-ATF5) or its control (Ad-Con) for 24 h. CHIP-qPCR assay was performed using antibodies against ATF5 or IgG (*n* = 5 independent experiments). **f** The mRNA (left panel) and protein (right panel) levels of INCA1 in TMEM11 KO and WT mice hearts (*n* = 6 mice per group). **g** The mRNA (left panel) and protein (right panel) levels of INCA1 in TMEM11 Tg and WT mice hearts (*n* = 6 mice per group). **h** Neonatal cardiomyocytes were infected with adenovirus harboring TMEM11, and transfected with si-NC or si-ATF5 for 48 h. The mRNA (left panel) and protein (right panel) levels of Inca1 were analyzed (*n* = 5 independent experiments). Data are presented as the mean ± s.d. One-way ANOVA (**h**), two-sided Student’s *t* test (**b**, **c**, **f** and **g**) or two-way ANOVA (**e**) was performed.

To explore the downstream targets of ATF5, we performed an RNA-seq analysis in ATF5-overexpressing cardiomyocytes (Fig. 7a and Supplementary Table 4). We screened for differentially expressed genes associated with proliferation regulation in the RNA-seq dataset. We validated their expression using qPCR. Among them, only the expression levels of *Inca1*, *Myog*, and *Fap* were significantly increased in ATF5-overexpressing cardiomyocytes compared to those in the control cells (Fig. 7b). Furthermore, changes in the expression levels of these three genes in ATF5-overexpressing cardiomyocytes prompted us to investigate whether TMEM11 also regulates their expression. Only *Inca1* expression was decreased in the hearts of TMEM11 KO mice compared to that in WT hearts (Supplementary Fig. 9a), indicating that *INCA1* might be a downstream target of the TMEM11-ATF5 pathway. In addition, previous studies have reported that INCA1 inhibits the interaction of CDK2 with cyclin A1, thereby causing a S to G2/M cell cycle arrest and inhibiting cell proliferation [42–44]. In addition, our results showed that knockdown of INCA1 promotes cardiomyocyte proliferation (Supplementary Fig. 9b, c). Therefore, *INCA1* was selected for further investigation.

Next, we demonstrated that ATF5 knockdown reduced *Inca1* mRNA and protein expression (Fig. 7c), indicating that the ATF5 transcription factor promoted *Inca1* expression. We also observed an ATF5-binding consensus motif in the promoter region of the *Inca1* gene (Fig. 7d) and verified ATF5 binding to this sequence using ChIP-qPCR (Fig. 7e). In addition, a marked enrichment of *Inca1* promoter region was observed following ATF5 overexpression (Fig. 7e). Consistent with these in vitro findings, *Inca1* mRNA and protein expression decreased in TMEM11 KO mouse hearts compared to that in WT hearts (Fig. 7f), whereas *Inca1* expression levels dramatically increased in the hearts of TMEM11 Tg mice (Fig. 7g). In cardiomyocytes, TMEM11 increased the levels of *Inca1* mRNA and protein, and these effects were attenuated by ATF5 knockdown (Fig. 7h and Supplementary Fig. 9d). Taken together, these results suggested that INCA1 acts as a downstream target of TMEM11/ATF5 pathway, which inhibits postnatal cardiomyocyte proliferation and regeneration.

## Discussion

There is currently no information about the molecular mechanisms responsible for the withdrawal of cell cycle and the non-proliferative growth of cardiomyocytes in mammals after birth. In this study, we identified a mitochondrial transmembrane protein, TMEM11, involved in the proliferation of cardiomyocytes, in both postnatal and adult mice. TMEM11 knockout promoted myocardial cell proliferation, reduced MI area, and improved cardiac function in adult mice. Overexpression of TMEM11 inhibited neonatal mouse cardiomyocyte proliferation and cardiac regeneration in mouse hearts. TMEM11 interacted with METTL1 and promoted the m^7^G methylation of *Atf5* mRNA, thereby enhancing its expression and that of its downstream target *Inca1* to ultimately suppress cardiomyocyte proliferation. Our study demonstrated that METTL1-dependent m^7^G RNA hypermethylation is required for cardiomyocyte proliferation; however, substantial expression of TMEM11 after birth intervenes in this event and suppresses cardiomyocyte proliferation.

Several studies have reported that cardiomyocyte proliferation is almost absent in the adult heart, and the death of cardiomyocytes and damage to cardiac function caused by MI are irreparable [45, 46]. Hippo and Wnt signaling pathways are known to regulate cardiomyocyte proliferation. Similarly, inhibition of the transcriptional coactivator Yap restricts cardiac growth and regeneration [12]. In addition, many studies have found that MI causes molecular changes in cardiac cells, resulting in mitochondrial dysfunction, which is the main cause of functional damage in cardiomyocytes [47–49]. A few mitochondrial transcription factors ameliorate mitochondrial defects and HF after MI [50]. Although the functional role of mitochondria in regulating MI is well-documented, the exact function of mitochondrial proteins in myocardial cell proliferation remains largely unclear. Our study focused on the role of the mitochondrial transmembrane protein, TMEM11, in myocardial cell proliferation and MI treatment. Silencing TMEM11 increased cardiomyocyte proliferation, improved cardiac function, and reduced infarct size.

Post birth, the expression of TMEM11 in the heart gradually increased and became abundant in adult mice. TMEM11 inhibited cardiomyocyte proliferation in adult mice. TMEM11 knockdown enhanced myocardial nuclear mitosis and cytokinesis in neonatal mouse cardiomyocytes. In addition, TMEM11 KO mice showed reduced infarct size after MI, restored cardiac function, and increased proliferation of adult mouse cardiomyocytes. Overexpression of TMEM11 in neonatal mouse cardiomyocytes inhibited cardiomyocyte proliferation both in vitro and in vivo. Thus, our data suggests that TMEM11 affects the proliferation of adult cardiomyocytes and that the inhibition of TMEM11 can promote adult cardiomyocyte proliferation and reduce infarct size. There is, however, no current understanding of how TMEM11 suppresses in neonatal hearts or how it is upregulated in young and adult hearts. Further research is needed to determine the mechanisms behind this phenomenon.

TMEM11 is primarily located in the mitochondria of cardiomyocytes. Previous studies have shown that TMEM11 is a molecular switch in the mitochondria that adapts to different physiological needs, affects mitochondrial morphology, and regulates mitochondrial phagocytosis and cell death. Other studies have shown that TMEM11 influences mitochondrial diameter and tubular shape [51, 52]. In this study, we investigated whether TMEM11 affects mitochondrial function. Our results showed that TMEM11 knockdown in cardiomyocytes did not change the mitochondrial membrane potential, which is an important indicator of mitochondrial activity. Furthermore, TMEM11 had no effects on apoptosis and autophagy. However, we cannot completely exclude the possibility that TMEM11 regulates cardiomyocyte proliferation by influencing mitochondrial function and energy metabolism. Further studies are required to dissect the effects of TMEM11 on mitochondrial energy metabolism and cardiomyocyte proliferation.

Hippo signal pathway is an evolutionarily conserved pathway, which is activated by a series of phosphorylation, such as MST1/2, SAV1, LATS1 and LATS2, which jointly phosphorylate YAP, thus regulating the proliferation of cardiac myocytes [53, 54]. We examined whether YAP is required for cardiomyocyte proliferation mediated by TMEM11 in this study, and results showed that TMEM11 had no effect on YAP expression levels. In cardiomyocytes, co-immunoprecipitation failed to detect an interaction between TMEM11 and YAP, indicating that YAP is not involved in TMEM11-mediated pathway. The results of our study demonstrate ATF5 as a target of TMEM11 during regulating the cardiomyocyte proliferation. It was reported that other molecules or pathways including Notch and Wnt/β-catenin also participate in the regulation of development, differentiation and regeneration [55–57]. According to MeRIP-seq and RNA-seq analyses, more than 500 genes were differentially expressed in TMEM11 Tg mouse hearts compared to those in WT hearts; some of these are involved in metabolism, cell division, and growth. These results suggest that other genes may also act as downstream targets of TMEM11 to regulate cardiomyocyte proliferation and warrant further studies.

As a transcription factor, ATF5 targets the mitochondria through its amino-terminal mitochondrial-targeting sequence in the absence of mitochondrial stress. However, during mitochondrial dysfunction, ATF5 cannot be imported into the mitochondria and is transported to the nucleus instead via its nuclear localization signal, inducing the transcription of genes that affect mitochondrial apoptosis inhibition, anti-apoptotic mechanisms, cell growth, and migration [58–63]. In addition, ATF5 has been shown to be a critical regulator of cell proliferation and survival [64–66]. Consistent with these findings, we found that ATF5 regulates cardiomyocyte proliferation. Our results showed that TMEM11 increased ATF5 expression, which promoted the binding of ATF5 to the promoter region of *Inca1* and induced the expression of INCA1. Enhanced INCA1 expression inhibited the transition from S to G2/M in the cell cycle, thus preventing cardiomyocytes from entering the cell cycle [65, 67].

The m^7^G modification exists within tRNAs, rRNAs, and mRNA caps and is implicated in nearly all aspects of the mRNA life cycle. A recent study verified that m^7^G modification mediated by METTL1 within mammalian mRNA affects mRNA translation [40], miRNA structure, and cell migration [41]. As a matter of fact, its role in cardiac development is unknown, especially when it comes to cardiac proliferation. Our study revealed that transcriptome-wide m^7^G sequencing analysis in TMEM11-overexpressing hearts revealed that METTL1-induced m^7^G hypermethylation of *Atf5* mRNA was related with enhanced translation of *Atf5* mRNA, which was evident from the remarkable increase in *Atf5* mRNA and protein levels. Consistent with a previous study [40], our data showed that m^7^G modification promoted mRNA translation. Here, we investigated the previously unrecognized function of the m^7^G modification in the regulation of cardiomyocyte proliferation and uncovered the underlying mechanisms of m^7^G modification-mediated cardiomyocyte proliferation and cardiac regeneration. This study suggests that a novel m^7^G modification-based therapeutic strategy may be effective for cardiac repair and regeneration.

## Supplementary information

Supplementary text

Supplementary figure 1

Supplementary figure 2

Supplementary figure 3

Supplementary figure 4

Supplementary figure 5

Supplementary figure 6

Supplementary figure 7

Supplementary figure 8

Supplementary figure 9

Supplementary Table 1

Supplementary Table 2

Supplementary Table 3

Supplementary Table 4

Original Data File

aj-checklist

## Data Availability

All data in this study are provided in the paper and Supplementary Materials.
