# Supplementary material for: TMEM11 regulates cardiomyocyte proliferation and cardiac repair via METTL1-mediated m7G methylation of ATF5 mRNA
Source: Cell Death Differ. 2023 Jun 7;30(7):1786–98. doi: 10.1038/s41418-023-01179-0 (PMC10307882; doi:10.1038/s41418-023-01179-0)
Supplement: Supplementary file 1 — Supplementary text [file 41418_2023_1179_MOESM1_ESM.docx]

**SUPPLEMENTARY MATERIAL**

**Mouse model of myocardial infarction.** Mice aged 6 to 8 weeks (C57BL/6) were selected for the experiments. Anesthesia was performed with 4% chloral hydrate intraperitoneal injection. The chest and armpit hair were surgically removed (fully exposing the surgical area), and the surgical area was disinfected with 75% ethanol. The ventilator was turned on, and all parameters (respiration rate 110 bpm) were set. Endotracheal intubation was performed in mice. Open the chest cavity between the third and fourth costal with ophthalmic scissors to fully expose the heart and the left anterior descending coronary artery (LAD) or the region. A 6-0 needle suture was taken with a needle holder, and the needle was inserted 2 mm at the lower margin of the left atrial appendage. The suture was passed through the LAD to block the blood flow of the LAD completely. After ligation, 4-0 sutures were used to completely close the thoracic opening (to ensure no gaps or misplacements) to close the thoracic cavity, and each layer of muscle and skin was sutured from inside to outside.

**Echocardiography measurement.** Echocardiography was performed using the Vevo2100 imaging system. We recorded two-dimensional-guided M-mode images in parasternal views (both short and long axis) at the level of papillary muscles to detect the left ventricle dimension. The fractional shortening (FS) was calculated using the standard equation.

**TTC staining, H/E and Masson.** Mice with myocardial infarction model were sacrificed, their hearts were quickly removed, placed in PBS solution, and frozen at -80℃ for 30 minutes to set. The heart sections, 2 mm thick, were placed in TTC staining solution for 30 minutes, and we washed the excess dye with PBS solution and took photographs immediately.

Paraffin sections were treated with xylene dewaxing for 5 minutes, anhydrous ethanol for 5 minutes, 90% ethanol for 5 minutes, 80% ethanol for 5 minutes, 70% ethanol for 5 minutes, and distilled water for 5 minutes. Hematoxylin was dyed for 5 minutes, we washed the excess dye with distilled water, and eosin was dyed for 30 seconds. Finally, it was dehydrated, transparent, and sealed.

The paraffin sections were dewaxed and stained with weigert for 5 minutes, acidic ethanol differentiation for 10 seconds, acidic ethanol was washed with distilled water, Masson cyaniding solution was stained for 2 minutes, Masson cyaniding solution was washed with distilled water, and fuchsin-red was stained for 5 minutes, we cleaned the weak acid solution for 1 minute, we cleaned phosphomolybdic acid for 2 minutes, we cleaned a weak acid solution for 2 minutes. Aniline blue staining for 1 min, weak acid solution cleaning for 2 min, dehydration, transparent, sealing.

**Nuclear/Cytosol Fractionation.** We used the BioVision kit for nucleocytoplasmic separation. First, we washed the cells with PBS and collected cardiomyocytes using a 4°C centrifuge at 600g/min for 5 min. Discard the supernatant and add 200 ul CEB-A (containing DDT and Protease Inhibitors), shake vigorously for 5 seconds, and leave on ice for 10 minutes. Add 11 ul CEB-B, shake vigorously for 5 seconds, and incubate on ice for 1 minute. Continue oscillating vigorously for 5 seconds. Centrifuge at 4°C, 16000g/min, 5 minutes. The supernatant was collected in a new Eppendorf tube and placed on ice. 100 ul NEB was added to the precipitate; the precipitate was violently shocked for 15 seconds and placed on the ice for 10 minutes. Centrifuge at 4°C, 16000g/min, for 10 minutes. The supernatant was transferred to a new eppendorf tube and stored in a refrigerator at -80℃.

**Mitochondrial isolation.** We used Solarbio to extract mitochondria from cardiomyocytes, the cardiomyocytes were washed in the petri dish with PBS and centrifuged at 800g for 5-10 min to collect the cells. Then the cells were re-suspended with 1.0 ml precooled Lysis Buffer. The cell suspension was transferred to a small-capacity glass homogenizer and ground in an ice bath 30 times. The cell homogenates were transferred to the centrifuge tube and centrifuged at 1000g for 5 min at 4℃. The supernatant was taken and transferred to a new centrifugal tube, centrifuged at 12,000 g at 4℃ for 10 min. The supernatant after centrifugation contained cytoplasmic components from which cytoplasmic proteins could be extracted. We transferred the supernatant to a new centrifuge tube, and mitochondria were deposited at the bottom of the tube. The mitochondrial precipitate was suspended by adding 0.5 mL Wash Buffer to the sediment, centrifuged at 1000 g at 4℃ for 5 min. The supernatant was taken and transferred to a new centrifugal tube, centrifuged at 12,000 g at 4℃ for 10 min. We discarded the supernatant, and high purity mitochondria precipitated at the bottom of the tube.

**Immunoprecipitation.** Mouse cardiomyocytes were extracted and transfected with adenovirus for 24 hours before cells were collected. RIPA (including PMSF) lysate was added and lysed on ice for 30 minutes, centrifuge at 4℃ at 12000rpm/min for 15 minutes. The supernatant was taken, and 1ug specific antibody was added. On the second day, 10ul protein A agarose beads were added and incubated for 4 hours. The non-specific binding was removed by cleaning 3 times with an appropriate lysis solution. Finally, we used an appropriate lysis solution for re-suspension, and we added protein loading buffer at 95℃ for 10 minutes. SDS-page electrophoresis and LC-MS/MS analysis.

**RNA****-binding protein immunoprecipitation (RIP) assay.** RIP assay was performed using the Megna RIP RNA-binding Protein Immunoprecipitation Kit (Millipore) according to the manufacturer’s instructions. In brief, the cells were lysed with RIP lysis buffer (50 mM Tris pH 8.0, 150 mM NaCl, 10% Glycerol, 1 mM EDTA, 50 mM NaF, 0.1% NP-40) containing protease and RNAase inhibitors. and the whole lysate (100 ml) incubated overnight with the primary antibodies or IgG (negative control) coated beads at 4°C. The protein-RNA complexes bound with beads were captured with magnetic protein A/G beads and centrifuged at 12000g for 5 min at 4°C. The supernatant was discarded, RNA-binding protein were washed, eluted and treated with Proteinase K. The purified RNA was extracted with Trizol reagent and qRT-PCR was conducted.

**Mitochondrial membrane potential assay.** We used the meilunbio kit to measure mitochondrial membrane potential. First, remove the cell culture medium and add 1ml of cell culture medium. Add 1ml JC-1 dye working solution and fully mix. Incubate in the cell incubator at 37℃ for 20 minutes. After incubation at 37 ℃, remove the supernatant, and wash it twice with JC-1 dye buffer (1**×**) in ice bath. Observation under laser confocal microscope.

**Mass spectrometry analysis.** Primary myocardial cells were extracted from neonatal mice (1-2 days old), the cardiomyocytes were infected with flag-TMEM11 adenovirus, followed by immunoprecipitation with anti-Flag M2 Magnetic beads (Sigma-Aldrich) to pull-down the binding protein of TMEM11. The flag-TMEM11 binding protein was extracted and the protein samples were subjected to SDS-PAGE gel electrophoresis and stained with coomassie brilliant blue. The extracted band was sent to a commercial facility for LC-MS/MS analysis (Shanghai Applied Protein Technology Co., LTD., Shanghai, China).

**Western Blot.** Total protein was extracted from cardiomyocytes or tissues by RIPA and PMSF. The BCA kit measured the concentration of total protein, and the enzyme reader read the OD value. Protein loading buffer was added to protein samples at 95℃ for 10min according to the concentration. The protein samples were transferred to the PVDF membrane by SDS-PAGE electrophoresis, and the protein expression was detected using specific antibodies. Antibodies were used as follows: TMEM11 (Proteintech, Cat: 16564-1-AP, 1:1500); GAPDH (ABclonal, Cat: A19056, 1:5000); Anti-ATF5 (abcam Cat: AB184923, 1:1000); INCA 1 (Bioss, BS-7899R, 1:1000).

**RT-qPCR.** TRIZOL reagent was used to extract total RNA from mouse cardiomyocytes or tissues, and we used nanodrop One to measure the concentration and purity of total RNA. The AG Evo M-MLV RT Kit reverses transcription Kit converted the total RNA into cDNA. We used SYBR Green Kit for fluorescent quantitative PCR. qPCR was performed by CFX96 real-time fluorescent quantitative PCR detection system. The following sequences of primers were used for qRT-PCR analysis: TMEM11 forward primer was 5’-AACGCCCAGGACCAGTTTG-3’; the reverse primer was 5’-GTGCTGTCTCGTCTCCAATTC-3’. ATF5 forward primer was 5’-TGGGCTGGCTCGTAGACTAT-3’; the reverse primer was 5’-GTCATCCAATCAGAGAAGCCG-3’. Inca1 forward primer was 5’-ATGCCTCAGCCGTATGGAGAT-3’; the reverse primer was 5’-GCCCTCAGAATTGGTGGAATGTA-3’. GAPDH forward primer was 5’-AGGTCGGTGTGAACGGATTTG-3’; the reverse primer was 5’-TGTAGACCATGTAGTTGAGGTCA-3’.

**Infection of adenovirus into cardiomyocytes.** Adenovirus was used for the overexpression and inhibition of TMEM11 in cell experiments. The adenovirus and their negative controls were obtained from Obio Technology Corp., Ltd (Shanghai, China). Primary myocardial cells were extracted from neonatal mice (1-2 days old)，the cardiomyocytes were transfected with adenovirus (5×10^10^ moi) for 4-6 h and then cultured in a new medium for 24h. The samples were collected for functional analysis by qPCR and western blot.

**Injection of AAV9 vectors in the adult mice.** Adeno-associated virus serotype 9 (AAV9) was used for the inhibition of ATF5 in the animal model. The AAV9-ATF5 and their negative controls were obtained from Hanbio Biotechnology Co., Ltd (Shanghai, China). For the experiments using ATF5 inhibition AAV9 virus, AAV9 viruses were diluted in PBS, 6-8 weeks C57BL/6J mice were treated with AAV9-control or AAV9-ATF5 (2×10^11^ vector genomes (vg) /mouse) by tail vein injection before MI. The hearts were collected at 60 days post-MI to perform the histological, echocardiography and functional analyses.

**Chromatin Immunoprecipitation (ChIP).** ChIP analysis were carried out using a chromatin immunoprecipitation assay kit (Millipore, Billerica, MA) and the experiment performed in accordance with the manufacturers protocol as we previously described^[1](#_ENREF_1)^. In brief, the primary cardiomyocytes were crosslinked with 1% formaldehyde (for 10 min) at room temperature and quenched with ice-cold 0.125M glycine for 5 min. Then the cells were centrifuged at 2500g for 2 min, washed with PBS, and resuspended in ice-cold cell lysis buffer (kept at 4°C for 1 h). The cell lysate was sonicated to generate chromatin fragments with an average length about 500–800 bp. The samples were precleared using protein A-agarose (Roche) for 1 h at 4°C followed by an overnight incubation with anti-Tfec or anti-mouse IgG at 4°C. The immune complexes were precipitated with protein A-agarose (pre-blocked with 2 μg/ml of salmon sperm DNA at 4 °C overnight) for 4 h. The precipitated DNA fragments were purified using a QIAquick Spin Kit (Qiagen) for PCR assay.

**mRNA-seq.** MRNA-Seq high throughput sequencing and subsequent bioinformatics analysis were done by Cloud-Seq Biotech (Shanghai, China). The total RNA was extracted from wild type and TMEM11 Tg mice hearts. Briefly, total RNA (1 μg) was used for removing the rRNAs using Ribo-Zero rRNA Removal Kits (Illumina, San Diego, CA, USA). RNA libraries were constructed by using rRNA-depleted RNAs with TruSeq Stranded Total RNA Library Prep Kit (Illumina, San Diego, CA, USA) according to the manufacturer’s instructions. Libraries were controlled for quality and quantified using the BioAnalyzer 2100 system (Agilent Technologies, Inc., USA). 10 pM libraries were denatured as single-stranded DNA molecules, captured on Illumina flow cells, amplified in situ as clusters and finally sequenced for 150 cycles on Illumina HiSeq Sequencer according to the manufacturer’s instructions. Paired-end reads were harvested from Illumina HiSeq 4000 sequencer, and were quality controlled by Q30. After 3’ adaptor-trimming and low quality reads removing by cutadapt software (v1.9.3), the high quality clean reads were aligned to the reference genome (UCSC MM10) with hisat2 software (v2.0.4). Then, guided by the Ensembl gtf gene annotation file, cuffdiff software (part of cufflinks) was used to get the gene level FPKM as the expression profiles of mRNA, and fold change and p-value were calculated based on FPKM, differentially expressed mRNA were identified.

**TUNEL apoptosis detection kit.** Resuspend cells in cell culture medium and suck 50-100 μl cell suspension was dropped onto a slide. Fix the cells, immerse the slide in polyformaldehyde containing 4% freshly prepared PBS, and place at 4 ℃ for 25 minutes. Wash the slide, immerse it in PBS, and leave it at room temperature for 5 minutes. Repeat washing with PBS once. Each sample can be immersed in 0.2% Triton X-100 solution and incubated at room temperature for 5 minutes for permeability treatment. PBS washed the sample three times. Dilute 5 with deionized water in a ratio of 1:5× Equilibration Buffer. Add 100 drops per sample μL 1 × Equip the buffer to completely cover the area of the sample to be tested, and incubate it at room temperature for 10-30 minutes. Use 50 μl TdT incubation buffer was dropwise added to the slide and incubated at 37℃ for 60 minutes. Finally, DAPI staining was performed.

**SUPPLEMENTAL FIGURE LEGENDS**

**Supplementary Figure 1.** **The subcellular localization and expression of TMEM11 in cardiomyocytes and its involvement in the regulation of cardiomyocyte proliferation. (a)** Distribution of TMEM11 was detected by immunofluorescence. Cardiomyocytes were stained with anti-TMEM11 antibody, and monitored by FITC-labeled secondary antibody (green) and DAPI (blue), and mitochondria were labeled with MitoTracker (red), Bar = 20 μm. **(b)** The expression level of TMEM11 in different tissues of adult mice as determined by western blot (n=3 independent experiments). **(c)** The expression level of TMEM11 in different types of cells in the mouse heart by western blot (n=3 independent experiments). **(d)** Neonatal mice cardiomyocytes were infected with adenovirus harbouring negative control (CTRL) or mouse TMEM11 gene (TMEM11) for 48 h. Representative confocal images of pH3-positive cardiomyocytes (Bar=20 μm). **(e)** Ki67-positive cardiomyocytes were measured in CTRL and TMEM11 treated cardiomyocytes (n=6 independent experiment). **(f)** Aurora-B-positive cardiomyocytes were measured in CTRL and TMEM11 treated cardiomyocytes (n=6 independent experiment). Data are presented as the mean ± s.d. Two-sided Student’s t-test **(e** and **f)** was performed.

**Supplementary Figure 2.** **TMEM11 has no effects on mitochondrial membrane potential, apoptosis and autophagy.** Neonatal mice cardiomyocytes were infected with adenovirus harbouring TMEM11-shRNA, TMEM11 or its control (TMEM11-shNC) for 48 h. **(a, b)** Mitochondrial membrane potential was assayed using the meilunbio kit. **(c)** Apoptosis was assayed using the TUNEL kit. (n=5 independent experiments). **(d)** The expression levels of Caspase 3 and LC3 were detected by western blot (n=4 independent experiment). Data are presented as the mean ± s.d. One-way ANOVA **(c)** was performed.

**Supplementary Figure 3.** **TMEM11 mediates cardiomyocytes proliferation in adult hearts. (a)** Representative echocardiographic images in WT and TMEM11 KO mice. **(b)** Echocardiography analysis of left ventricular function (ejection fraction, EF%) after MI (n=7 mice). **(c)** Left ventricular muscle sections stained with wheat germ agglutinin (WGA) to demarcate cell boundaries (Bar=20 μm). **(d)** Quantitative analysis represents measurement of the cross-section of cardiomyocytes in multiple fields from 6 independent samples per group. **(e)** Quantification of the number of cardiomyocytes from WT and TMEM11 KO mice (n=3–4 fields from 6 hearts). **(f, g)** The expression of cardiac hypertrophy markers *Bnp* and *Myh7* was analyzed in adult WT and KO mice (n=6 mice per group). Data are presented as the mean ± s.d. Two-sided Student’s t-test **(b** and **d**-**g)** was performed.

**Supplementary Figure 4.** **TMEM11 mediates cardiac repair in adult hearts after ischemic injury. (a)** Scheme of the myocardial infarction (MI) experiments in mice and time points of sample collections for various analyses. **(b)** Representative echocardiographic images in WT and TMEM11 KO mice subjected to MI. **(c)** Echocardiography analysis of left ventricular function (ejection fraction, EF%) after MI (n=7 mice). **(d)** The expression level of TMEM11 in sham and MI by western blot (n=3 independent experiments). **(e)** The expression level of TMEM11 in sham and TAC by western blot (n=3 independent experiments). Data are presented as the mean ± s.d. Two-sided Student’s t-test **(c)** was performed.

**Supplementary Figure 5.** **TMEM11 overexpression inhibits cardiomyocytes proliferation. (a)** Western blot image (top) and statistical data (bottom) showing the expression level of TMEM11 in WT and TMEM11 Tg mice hearts (n=7 mice per group). **(b)** The expression of cardiac hypertrophy markers *Bnp* was analyzed in adult WT and Tg mice (n=5 mice per group). **(c)** The expression of cardiac hypertrophy markers *Myh7* was analyzed in adult WT and Tg mice (n=6 mice per group). **(d)** Echocardiography analysis of left ventricular function (ejection fraction, EF%) after MI (n=7 mice). **(e)** WT and TMEM11 Tg neonatal mice (P1) were subjected to MI. Representative confocal images of Ki67-positive cardiomyocytes in heart sections at 7 days after MI. **(f)** Representative confocal images of pH3-positive cardiomyocytes in heart sections at 7 days after MI. **(g)** Echocardiography analysis of left ventricular function (ejection fraction, EF%) in heart sections at 7 days after MI (n=7 mice). Data are presented as the mean ± s.d. Two-sided Student’s t-test (**a-d, and g**) was performed.

**Supplementary Figure 6.** **Epitranscriptome and transcriptome analyses in TMEM11 Tg** **mice hearts.** **(a)** LC-MS/MS analysis in Flag-TMEM11 immunopurified protein complex identified METTL1. **(b)** MeRIP-seq was performed in TMEM11 Tg and WT mice hearts. Pie chart depicting the distribution of m^7^G peaks in different transcript segments in TMEM11 Tg and WT mice hearts. **(c** and **d)** Gene ontology (GO) analysis of enriched terms in biological processes associated with TMEM11 target genes.

**Supplementary Figure 7.** **TMEM11 promotes ATF5 expression by METTL1. (a)** Cardiomyocytes were transfected with ATF5-siRNA (si-ATF5) and its control (si-NC). ATF5 protein and mRNA levels were evaluated by western blot and qRT-PCR (n=6 independent experiments). **(b)** Neonatal cardiomyocytes were infected with adenovirus harbouring TMEM11, and transfected with si-NC or si-ATF5 for 48 h. Representative confocal images of pH3-positive cardiomyocytes (Bar=20 μm). **(c-e)** AAV9 loaded with ATF5 shRNA (sh-ATF5) or its control (sh-CTRL) was administered to adult WT mice. **(c)** The expression level of ATF5 in the mouse heart by western blot (n=3 independent experiments). **(d)** Echocardiography analysis of fractional shortening (FS, n=6 mice per group). **(e)** Quantification of the left ventricle infarct size measured as the percentage of left ventricle area at 8 weeks post-MI (n= 6 mice per group). INF, Infarct area. **(f)** The expression level of ATF5 in sham and MI by western blot (n=3 independent experiments). **(g)** The expression level of ATF5 in sham and TAC by western blot (n=3 independent experiments). **(h)** RIP-qPCR analysis in WT or TMEM11 KO mice hearts shows the level of *Atf5* mRNA binding to METTL1 (n=6 mice per group). **(i)** Neonatal cardiomyocytes were infected with adenovirus harbouring METTL1, TMEM11 or its control (NC) for 48 h. M^7^G modification level in *Atf5* mRNA was analyzed by MeRIP-qPCR and protein levels were evaluated by western blot (n=5 independent experiments). Data are presented as the mean ± s.d. One-way ANOVA **(a, d**, **e,** and **i)** or two-way ANOVA **(h)** was performed.

**Supplementary Figure 8.** **TMEM11 mediates the expression of ATF5 via METTL1.** Adenovirus harbouring METTL1 shRNA (sh-METTL1) or its control (sh-NC) was administered to adult TMEM11 mice, and heart samples were collected. **(a)** M^7^G modification level in *Atf5* mRNA was analyzed by MeRIP-qPCR (n=5 independent experiments). **(b)** The mRNA levels of *Atf5* were evaluated by qRT-PCR (n=6 independent experiments). **(c)** Cardiomyocytes were infected with adenovirus harbouring TMEM11, sh-METTL1 or its control (sh-NC) for 48 h. *Atf5* expression were evaluated by qRT-PCR (n=5 independent experiments). **(d)** The expression level of YAP in WT and KO mice by western blot (n=3 independent experiments). **(e)** The expression level of YAP in WT and Tg mice by western blot (n=3 independent experiments). **(f)** Immunoprecipitation (IP) of YAP were performed in the whole cell lysate of mouse cardiomyocytes using specific antibodies and resulting complex were purified and subjected to immunoblot analysis using YAP or TMEM11 antibodies. The whole cell lysate used as an input reference (n=3 independent experiment). Data are presented as the mean ± s.d. One-way ANOVA **(a-c)** was performed.

**Supplementary Figure 9.** **INCA1 promotes cardiomyocyte proliferation through TMEM11/ATF5. (a)** Expression levels of *Inca1, Myog* and *Fap* mRNA in TMEM11 KO and WT mice hearts (n=5 mice per group). Cardiomyocytes were transfected with INCA1-siRNA (si-INCA1) and its control (si-NC). **(b)** Ki67-positive cardiomyocytes were measured in si-NC and si-INCA1 treated cardiomyocytes (n=5 independent experiment). **(c)** pH3-positive cardiomyocytes were measured in si-NC and si-INCA1 treated cardiomyocytes (n=5 independent experiment). **(d)** Neonatal cardiomyocytes were infected with adenovirus harbouring TMEM11, and transfected with si-NC or si-INCA1 for 48 h. The expression level of ATF5 by western blot (n=3 independent experiments). Data are presented as the mean ± s.d. Two-sided Student’s t-test **(a-c)** was performed.
