# Supplementary material for: TMEM11 regulates cardiomyocyte proliferation and cardiac repair via METTL1-mediated m7G methylation of ATF5 mRNA
Source: Cell Death Differ. 2023 Jun 7;30(7):1786–98. doi: 10.1038/s41418-023-01179-0 (PMC10307882; doi:10.1038/s41418-023-01179-0)
Supplement: Supplementary file 2 — Supplementary figure 1 [file 41418_2023_1179_MOESM2_ESM.pptx]

## Slide 1
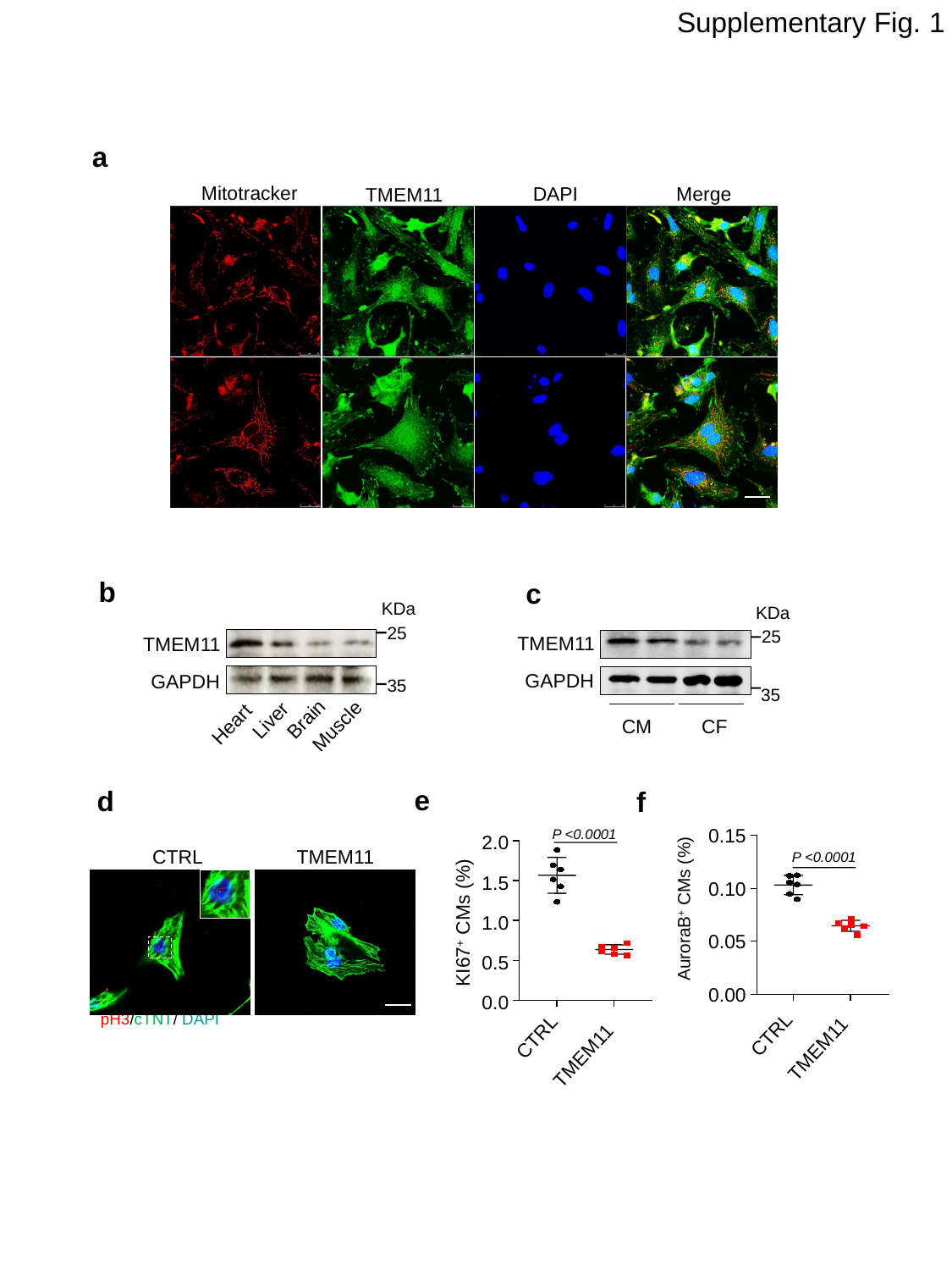

Supplementary Fig. 1
a
Mitotracker
Merge
DAPI
TMEM11
b
c
KDa
25
35
TMEM11
GAPDH
Brain
Liver
Muscle
Heart
KDa
25
35
TMEM11
GAPDH
CM
CF
e
d
CTRL
TMEM11
pH3/cTNT/ DAPI
f
AuroraB+ CMs (%)
0.15
P <0.0001
0.10
0.05
0.00
CTRL
TMEM11
P <0.0001
2.0
KI67+ CMs (%)
1.5
1.0
0.5
0.0
CTRL
TMEM11
