# Supplementary material for: TMEM11 regulates cardiomyocyte proliferation and cardiac repair via METTL1-mediated m7G methylation of ATF5 mRNA
Source: Cell Death Differ. 2023 Jun 7;30(7):1786–98. doi: 10.1038/s41418-023-01179-0 (PMC10307882; doi:10.1038/s41418-023-01179-0)
Supplement: Supplementary file 3 — Supplementary figure 2 [file 41418_2023_1179_MOESM3_ESM.pptx]

## Slide 1
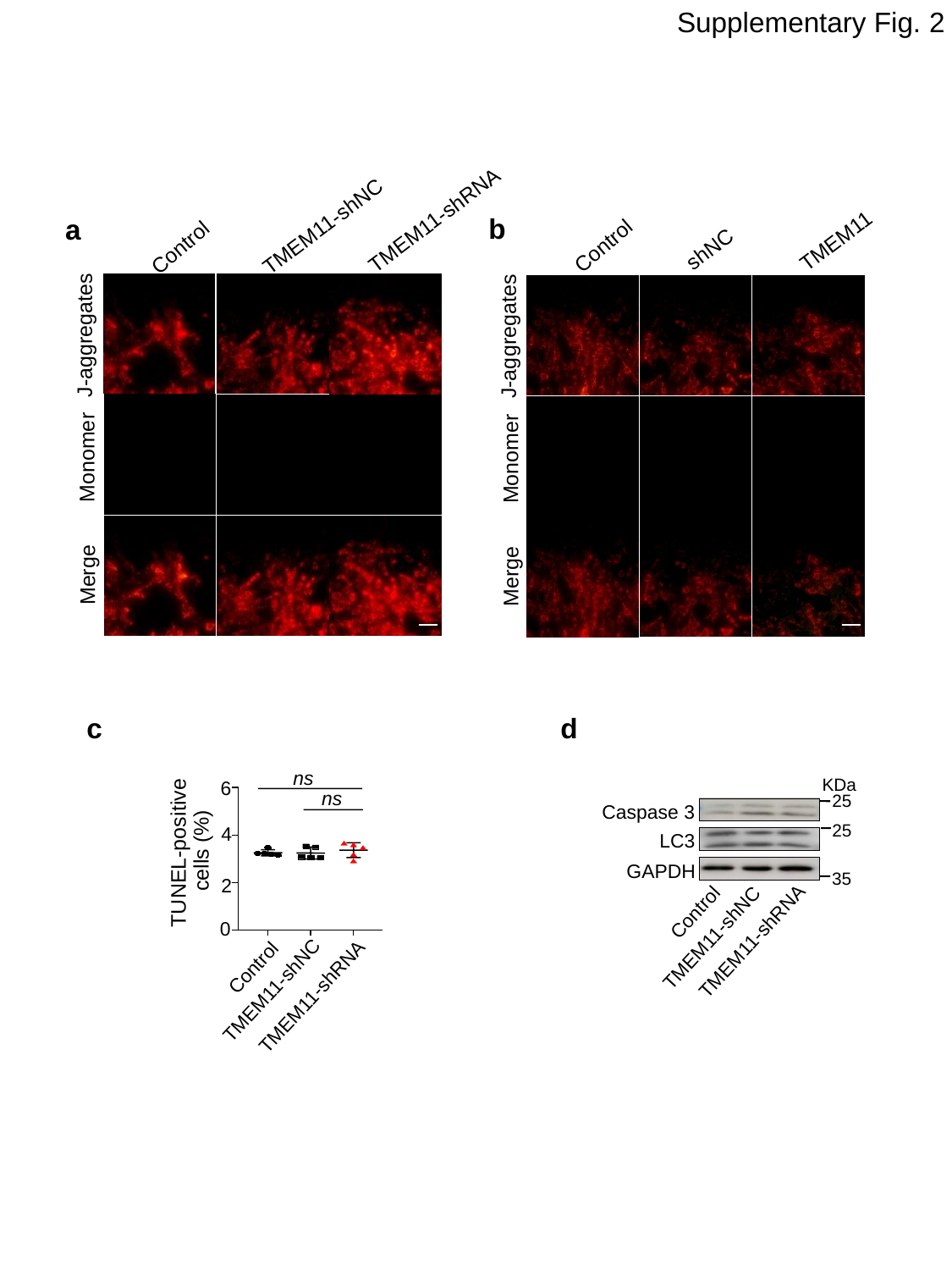

Supplementary Fig. 2
shNC
TMEM11-shRNA
TMEM11-shNC
Control
J-aggregates
Monomer
Merge
b
a
Control
TMEM11
J-aggregates
Monomer
Merge
 cells (%)
ns
6
ns
4
2
0
Control
TMEM11-shNC
TMEM11-shRNA
c
TUNEL-positive
d
KDa
25
Caspase 3
25
LC3
GAPDH
Control
TMEM11-shNC
TMEM11-shRNA
35
