# Supplementary material for: TMEM11 regulates cardiomyocyte proliferation and cardiac repair via METTL1-mediated m7G methylation of ATF5 mRNA
Source: Cell Death Differ. 2023 Jun 7;30(7):1786–98. doi: 10.1038/s41418-023-01179-0 (PMC10307882; doi:10.1038/s41418-023-01179-0)
Supplement: Supplementary file 4 — Supplementary figure 3 [file 41418_2023_1179_MOESM4_ESM.pptx]

## Slide 1
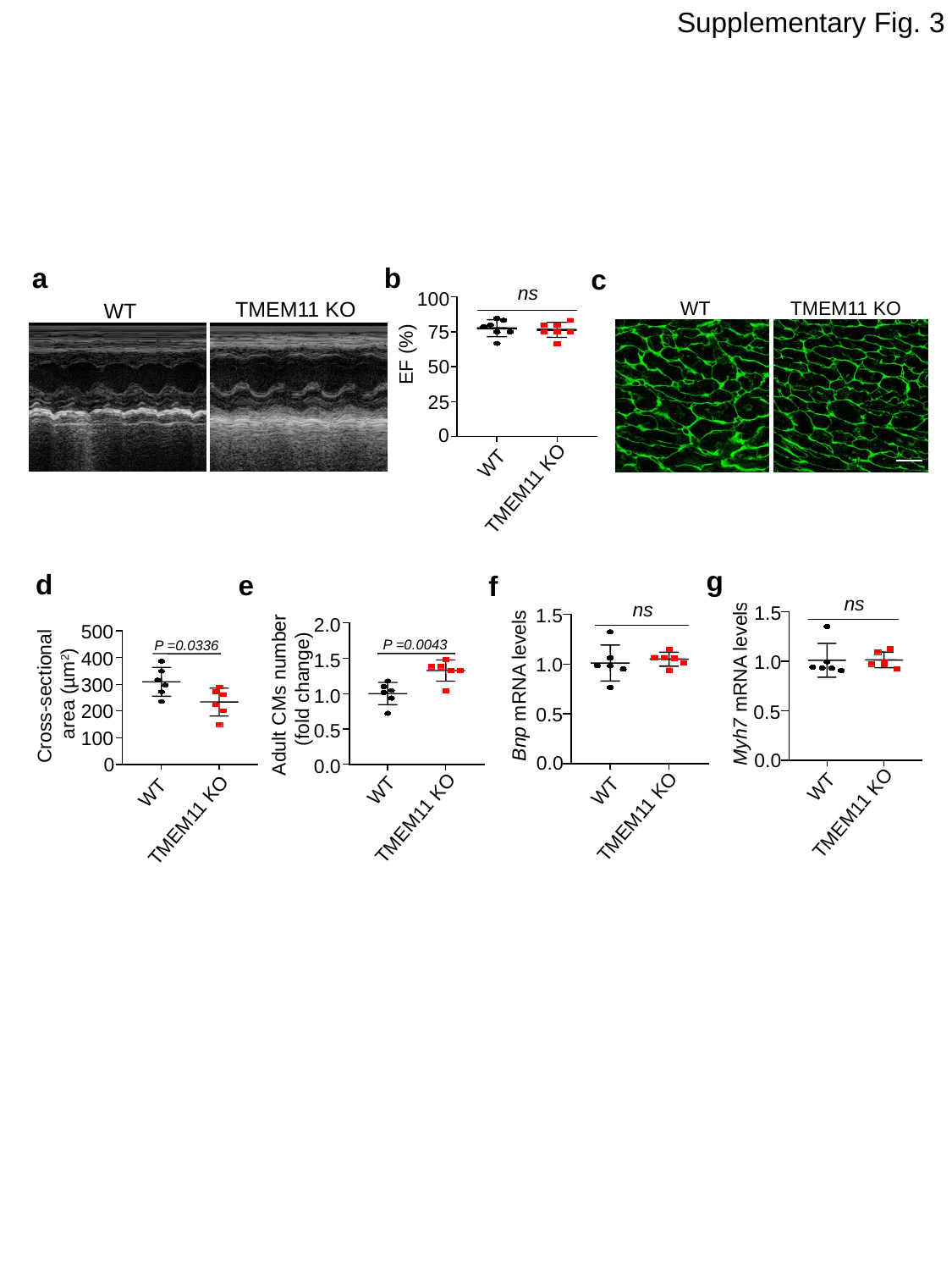

Supplementary Fig. 3
a
b
c
ns
100
EF (%)
75
50
25
0
WT
TMEM11 KO
TMEM11 KO
WT
TMEM11 KO
WT
g
d
e
f
ns
1.5
1.0
Myh7 mRNA levels
0.5
0.0
WT
TMEM11 KO
ns
2.0
P =0.0043
1.5
1.0
0.5
0.0
 Adult CMs number
 (fold change)
WT
TMEM11 KO
1.5
1.0
0.5
0.0
Bnp mRNA levels
WT
TMEM11 KO
500
400
300
200
100
0
P =0.0336
 Cross-sectional
 area (µm2)
WT
TMEM11 KO
