# Supplementary material for: TMEM11 regulates cardiomyocyte proliferation and cardiac repair via METTL1-mediated m7G methylation of ATF5 mRNA
Source: Cell Death Differ. 2023 Jun 7;30(7):1786–98. doi: 10.1038/s41418-023-01179-0 (PMC10307882; doi:10.1038/s41418-023-01179-0)
Supplement: Supplementary file 5 — Supplementary figure 4 [file 41418_2023_1179_MOESM5_ESM.pptx]

## Slide 1
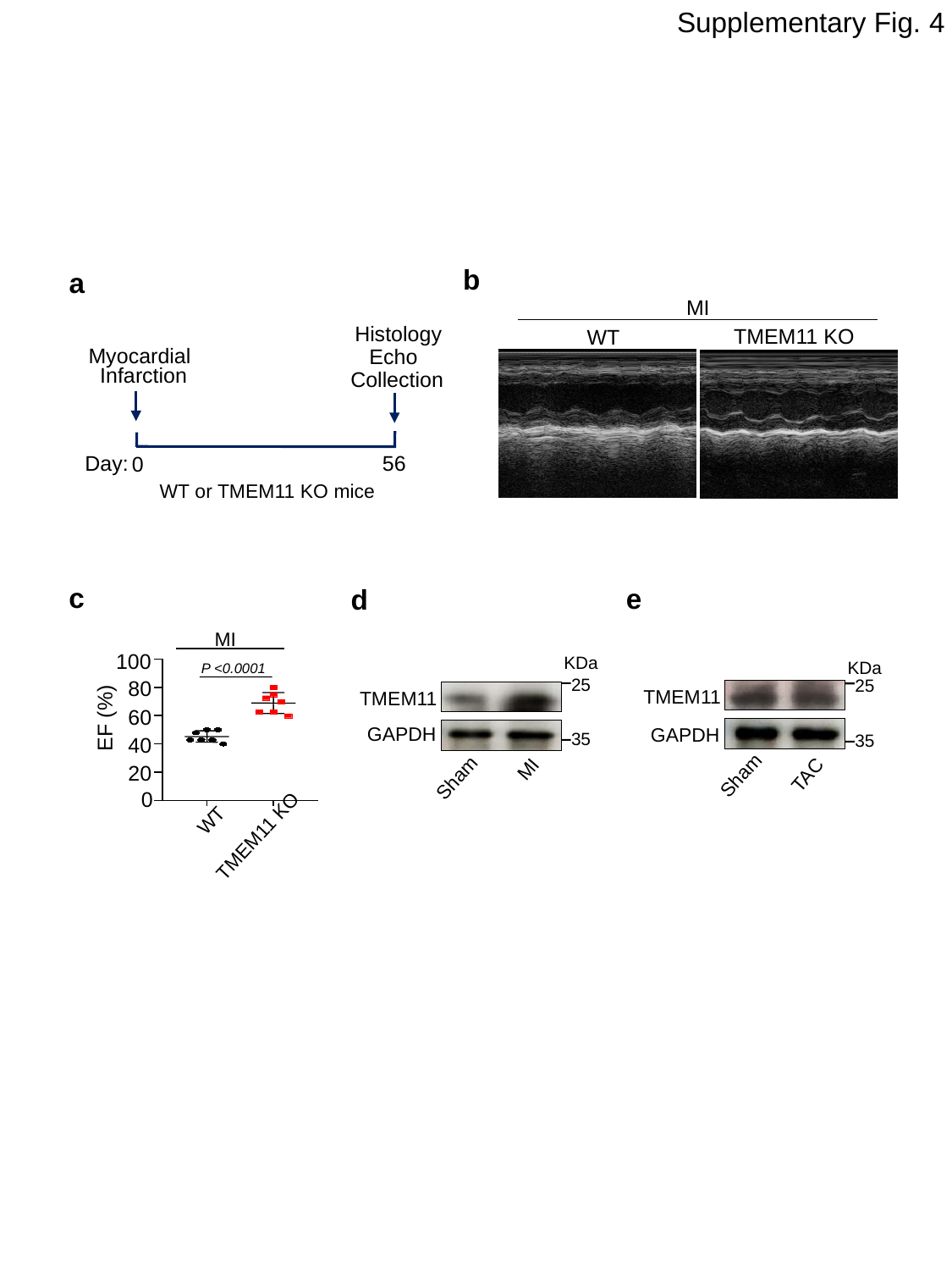

Supplementary Fig. 4
b
a
MI
TMEM11 KO
WT
Histology
Echo
Collection
Myocardial
Infarction
56
Day:
0
WT or TMEM11 KO mice
c
e
d
MI
100
P <0.0001
EF (%)
80
60
40
20
0
WT
TMEM11 KO
KDa
25
TMEM11
GAPDH
35
MI
Sham
KDa
25
TMEM11
GAPDH
35
TAC
Sham
