# Supplementary material for: TMEM11 regulates cardiomyocyte proliferation and cardiac repair via METTL1-mediated m7G methylation of ATF5 mRNA
Source: Cell Death Differ. 2023 Jun 7;30(7):1786–98. doi: 10.1038/s41418-023-01179-0 (PMC10307882; doi:10.1038/s41418-023-01179-0)
Supplement: Supplementary file 6 — Supplementary figure 5 [file 41418_2023_1179_MOESM6_ESM.pptx]

## Slide 1
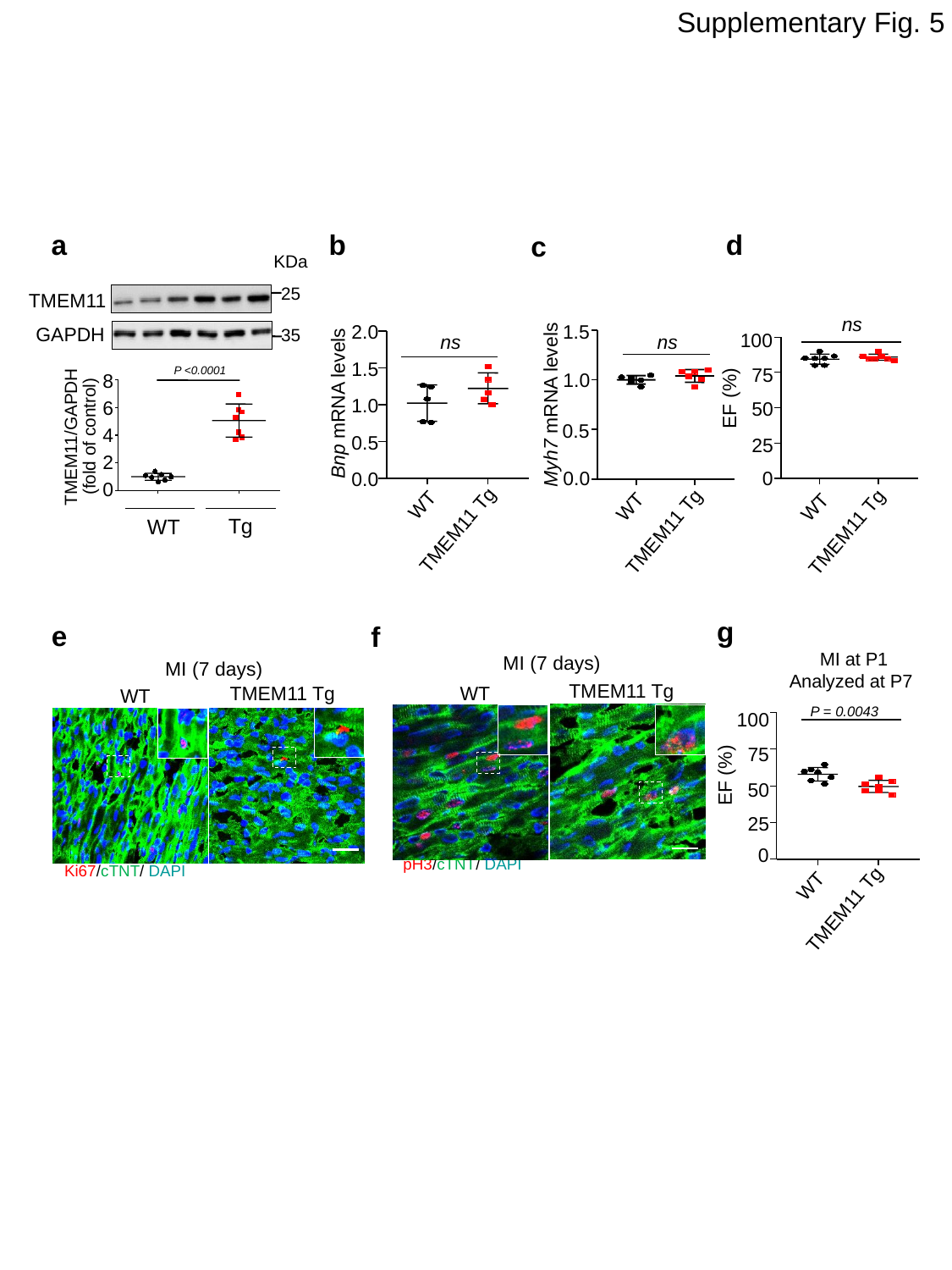

Supplementary Fig. 5
d
a
KDa
25
35
TMEM11
GAPDH
P <0.0001
8
6
4
2
0
Tg
WT
b
c
 TMEM11/GAPDH
 (fold of control)
ns
100
75
50
25
0
WT
TMEM11 Tg
1.5
1.0
Myh7 mRNA levels
0.5
0.0
WT
TMEM11 Tg
ns
2.0
1.5
1.0
0.5
0.0
ns
EF (%)
 Bnp mRNA levels
WT
TMEM11 Tg
g
e
f
 MI at P1
Analyzed at P7
P = 0.0043
100
EF (%)
75
50
25
0
WT
TMEM11 Tg
MI (7 days)
TMEM11 Tg
WT
pH3/cTNT/ DAPI
MI (7 days)
TMEM11 Tg
WT
Ki67/cTNT/ DAPI
