# Supplementary material for: TMEM11 regulates cardiomyocyte proliferation and cardiac repair via METTL1-mediated m7G methylation of ATF5 mRNA
Source: Cell Death Differ. 2023 Jun 7;30(7):1786–98. doi: 10.1038/s41418-023-01179-0 (PMC10307882; doi:10.1038/s41418-023-01179-0)
Supplement: Supplementary file 7 — Supplementary figure 6 [file 41418_2023_1179_MOESM7_ESM.pptx]

## Slide 1
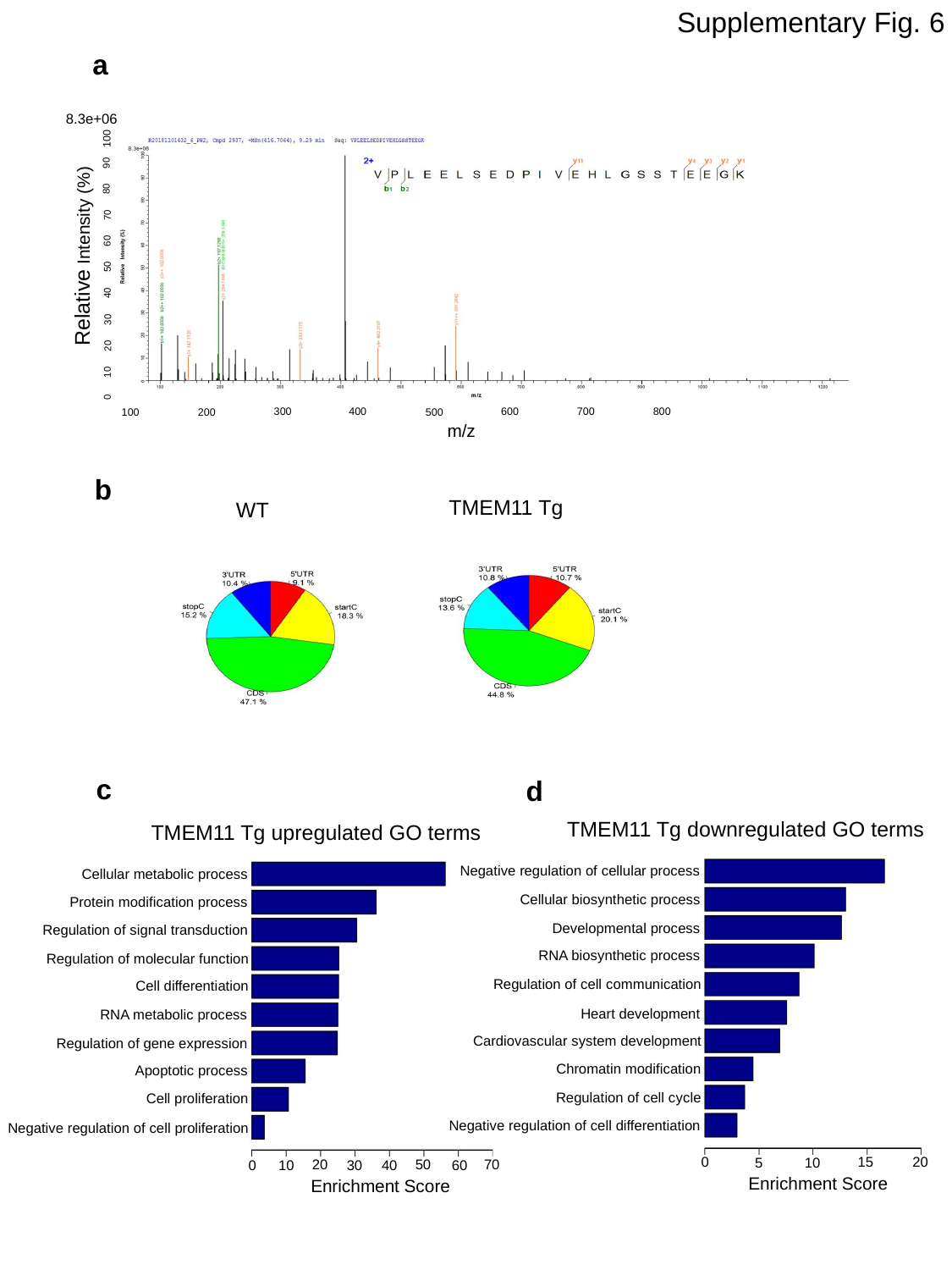

Supplementary Fig. 6
a
8.3e+06
100
90
80
70
60
 Relative Intensity (%)
50
40
30
20
10
0
700
800
600
300
400
100
500
200
 m/z
b
TMEM11 Tg
WT
c
d
TMEM11 Tg downregulated GO terms
TMEM11 Tg upregulated GO terms
Negative regulation of cellular process
Cellular biosynthetic process
Developmental process
RNA biosynthetic process
Regulation of cell communication
Heart development
Cardiovascular system development
Chromatin modification
Regulation of cell cycle
Negative regulation of cell differentiation
20
0
15
10
5
Enrichment Score
Cellular metabolic process
Protein modification process
Regulation of signal transduction
Regulation of molecular function
Cell differentiation
RNA metabolic process
Regulation of gene expression
Apoptotic process
Cell proliferation
Negative regulation of cell proliferation
20
50
70
60
40
0
10
30
Enrichment Score
