# Supplementary material for: TMEM11 regulates cardiomyocyte proliferation and cardiac repair via METTL1-mediated m7G methylation of ATF5 mRNA
Source: Cell Death Differ. 2023 Jun 7;30(7):1786–98. doi: 10.1038/s41418-023-01179-0 (PMC10307882; doi:10.1038/s41418-023-01179-0)
Supplement: Supplementary file 8 — Supplementary figure 7 [file 41418_2023_1179_MOESM8_ESM.pptx]

## Slide 1
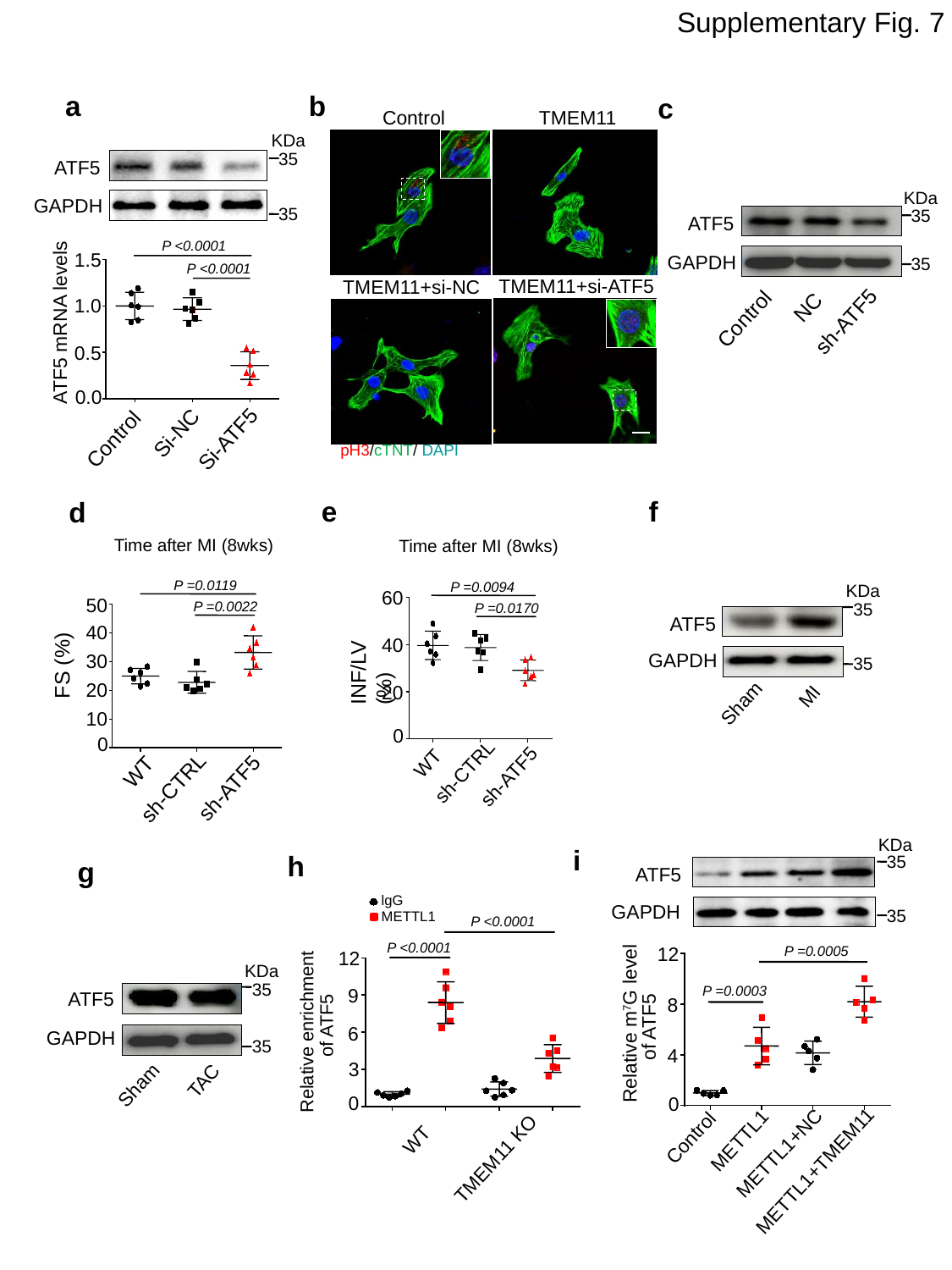

Supplementary Fig. 7
b
a
c
Control
TMEM11
TMEM11+si-ATF5
TMEM11+si-NC
pH3/cTNT/ DAPI
KDa
35
35
ATF5
GAPDH
KDa
35
ATF5
GAPDH
35
NC
Control
sh-ATF5
P <0.0001
1.5
P <0.0001
1.0
 ATF5 mRNA levels
0.5
0.0
Si-NC
Si-ATF5
Control
f
e
d
Time after MI (8wks)
P =0.0119
P =0.0022
50
FS (%)
40
30
20
10
0
WT
sh-CTRL
sh-ATF5
Time after MI (8wks)
P =0.0094
60
P =0.0170
INF/LV (%)
40
20
0
WT
sh-CTRL
sh-ATF5
KDa
35
ATF5
GAPDH
35
MI
Sham
KDa
35
35
ATF5
GAPDH
of ATF5
Relative enrichment
lgG
METTL1
P <0.0001
P <0.0001
12
9
6
3
0
WT
TMEM11 KO
i
h
g
Relative m7G level
of ATF5
P =0.0005
P =0.0003
12
8
4
0
METTL1
Control
 METTL1+NC
METTL1+TMEM11
KDa
35
ATF5
GAPDH
35
TAC
Sham
