# Supplementary material for: TMEM11 regulates cardiomyocyte proliferation and cardiac repair via METTL1-mediated m7G methylation of ATF5 mRNA
Source: Cell Death Differ. 2023 Jun 7;30(7):1786–98. doi: 10.1038/s41418-023-01179-0 (PMC10307882; doi:10.1038/s41418-023-01179-0)
Supplement: Supplementary file 9 — Supplementary figure 8 [file 41418_2023_1179_MOESM9_ESM.pptx]

## Slide 1
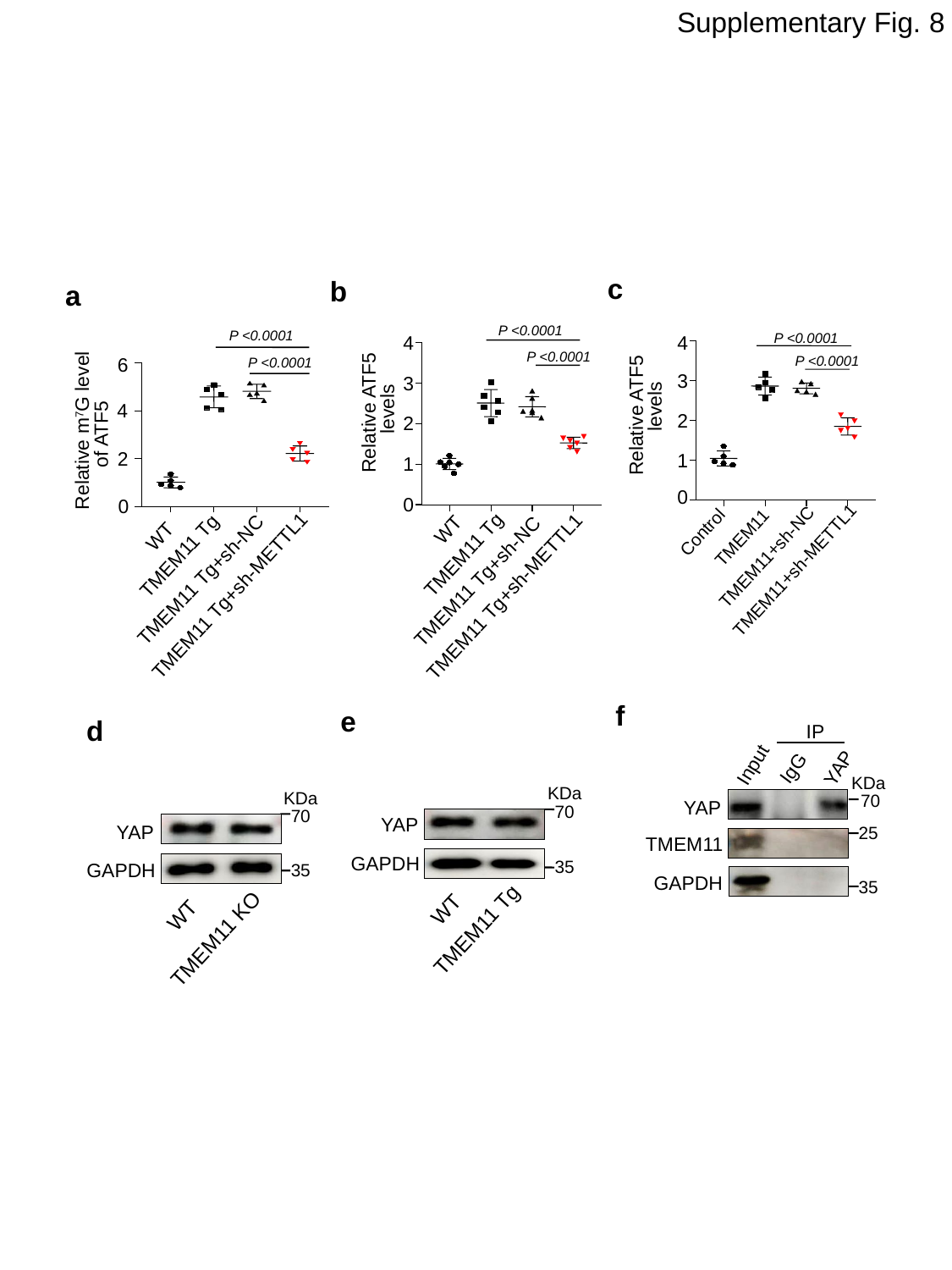

Supplementary Fig. 8
Relative ATF5
levels
b
P <0.0001
4
3
2
1
0
P <0.0001
WT
TMEM11 Tg
 TMEM11 Tg+sh-NC
TMEM11 Tg+sh-METTL1
Relative ATF5
P <0.0001
4
P <0.0001
levels
3
2
1
0
Control
TMEM11
TMEM11+sh-NC
TMEM11+sh-METTL1
Relative m7G level
of ATF5
P <0.0001
6
P <0.0001
4
2
0
WT
TMEM11 Tg
 TMEM11 Tg+sh-NC
TMEM11 Tg+sh-METTL1
e
c
a
IP
YAP
IgG
Input
YAP
TMEM11
GAPDH
f
d
KDa
70
KDa
70
35
YAP
GAPDH
WT
TMEM11 Tg
KDa
70
YAP
GAPDH
35
WT
TMEM11 KO
25
35
