# Supplementary material for: TMEM11 regulates cardiomyocyte proliferation and cardiac repair via METTL1-mediated m7G methylation of ATF5 mRNA
Source: Cell Death Differ. 2023 Jun 7;30(7):1786–98. doi: 10.1038/s41418-023-01179-0 (PMC10307882; doi:10.1038/s41418-023-01179-0)
Supplement: Supplementary file 10 — Supplementary figure 9 [file 41418_2023_1179_MOESM10_ESM.pptx]

## Slide 1
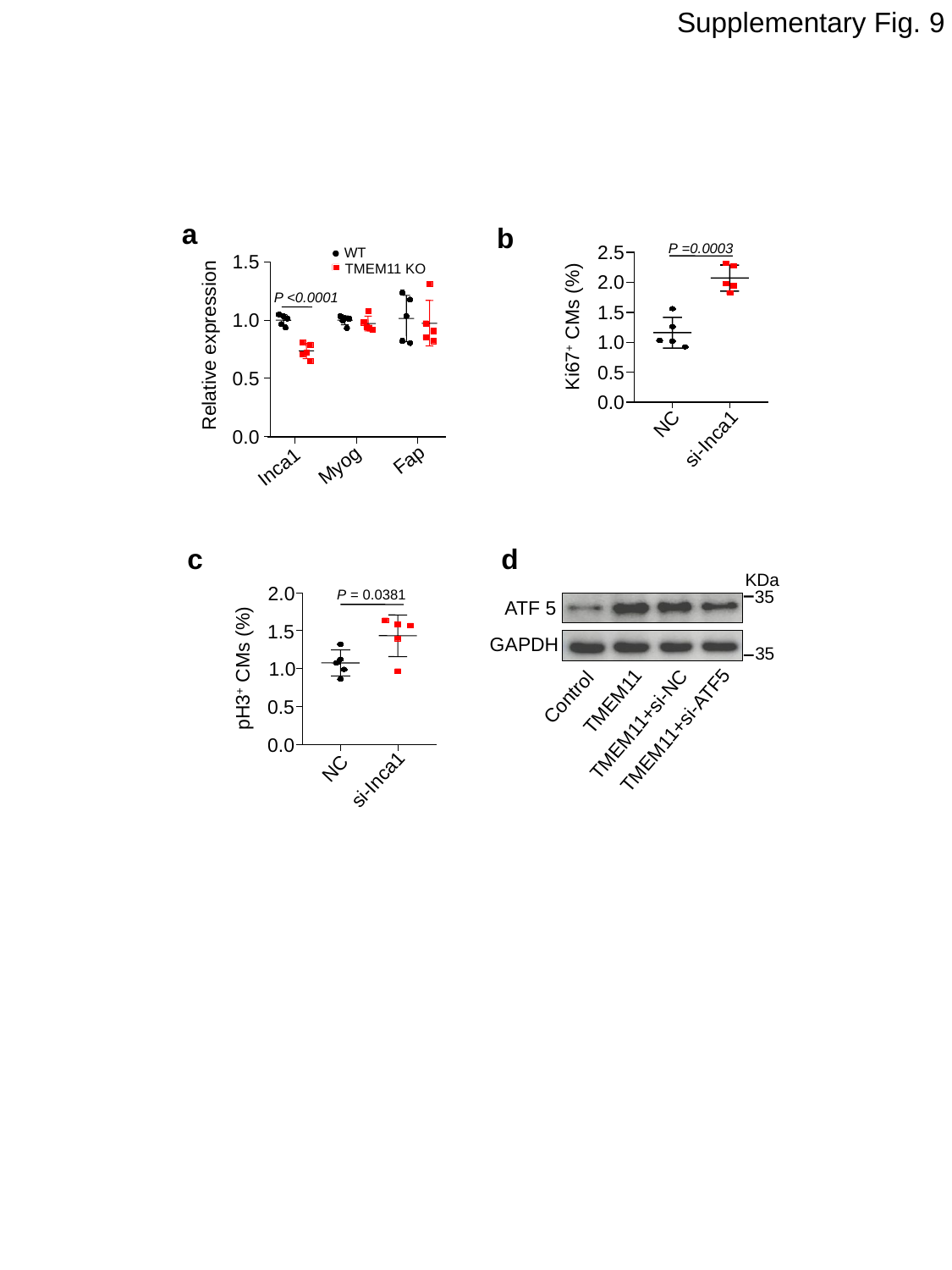

Supplementary Fig. 9
a
b
Ki67+ CMs (%)
P =0.0003
2.5
2.0
1.5
1.0
0.5
0.0
NC
si-Inca1
WT
TMEM11 KO
Relative expression
1.5
1.0
0.5
0.0
Fap
Myog
Inca1
P <0.0001
c
d
KDa
35
ATF 5
Control
TMEM11
TMEM11+si-NC
TMEM11+si-ATF5
GAPDH
35
pH3+ CMs (%)
2.0
P = 0.0381
1.5
1.0
0.5
0.0
NC
si-Inca1
