# Supplementary material for: TMEM11 regulates cardiomyocyte proliferation and cardiac repair via METTL1-mediated m7G methylation of ATF5 mRNA
Source: Cell Death Differ. 2023 Jun 7;30(7):1786–98. doi: 10.1038/s41418-023-01179-0 (PMC10307882; doi:10.1038/s41418-023-01179-0)

Figure 1 a

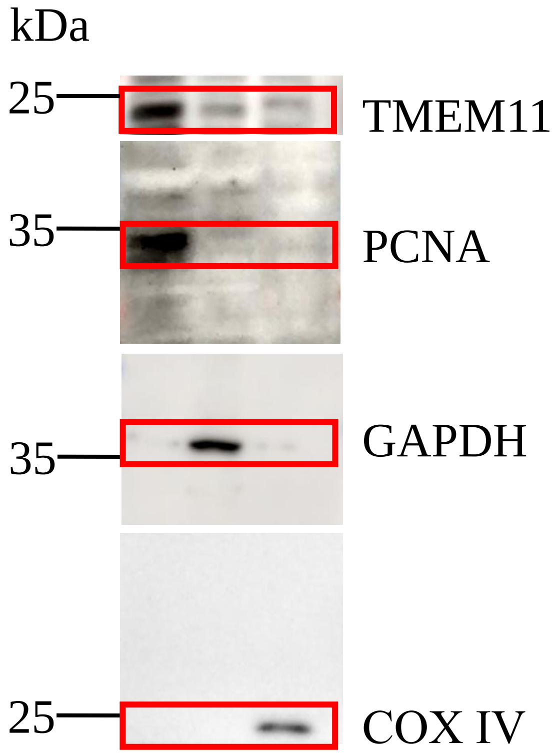

Figure 1 b

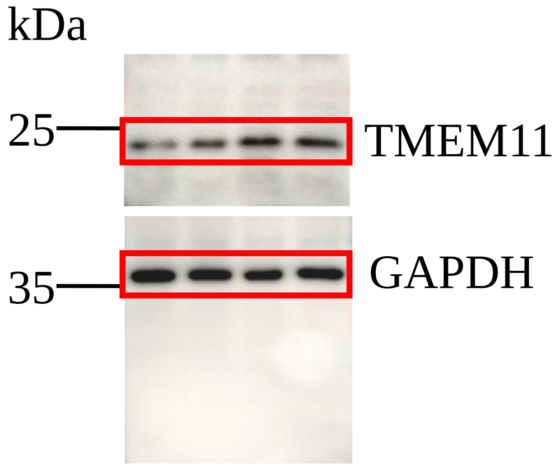

Figure 1 c

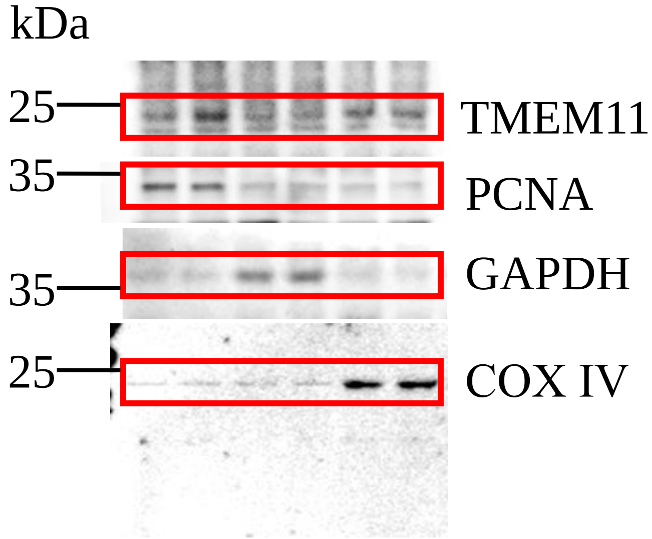

Figure 1 d

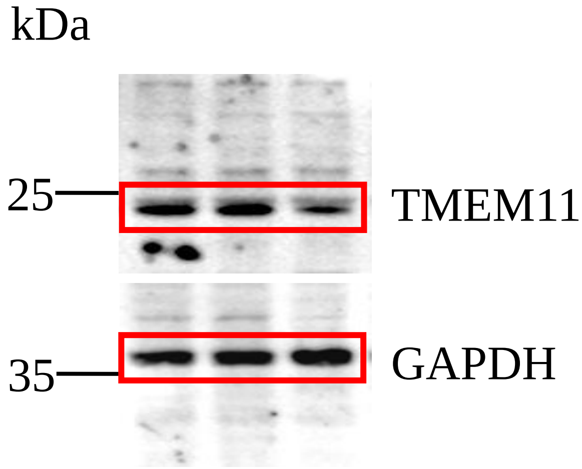

Figure 1 i

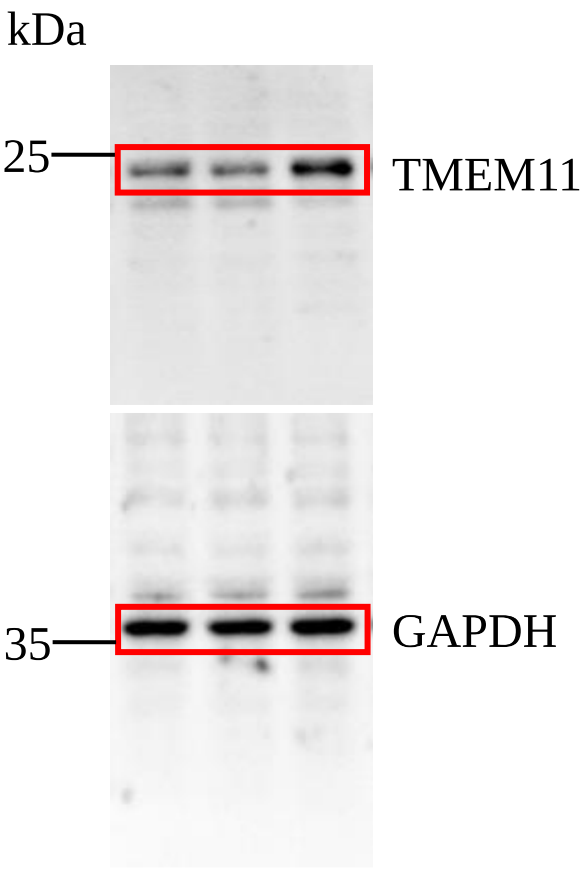

Figure 2 a

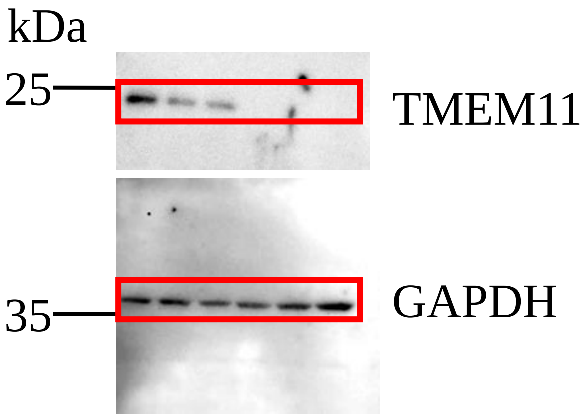

Figure 5 a

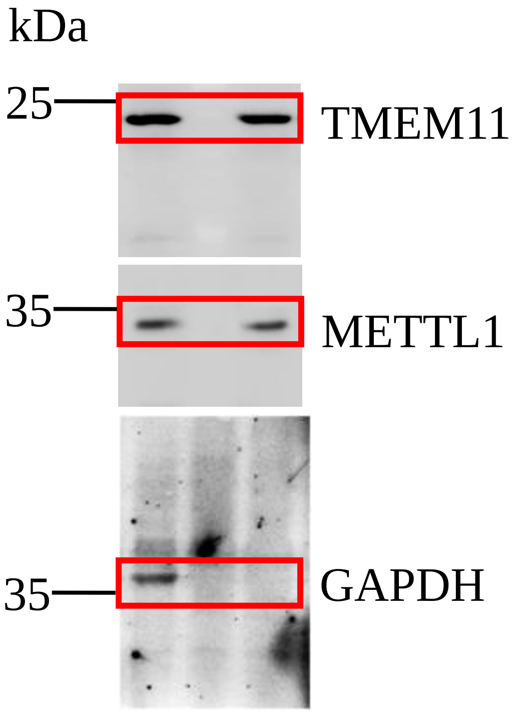

Figure 5 b

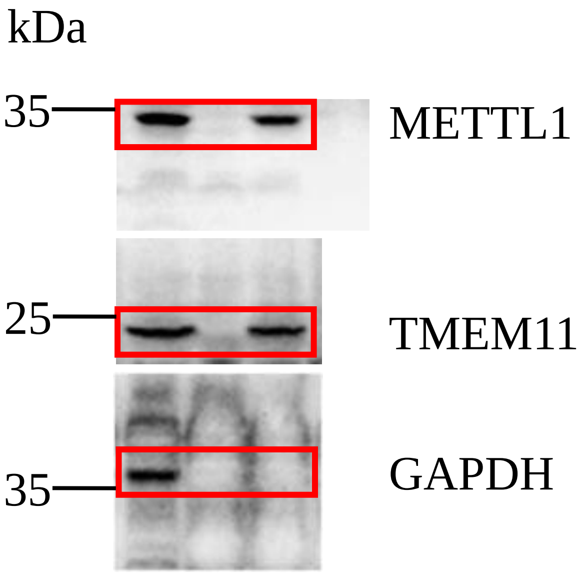

Figure 6 b

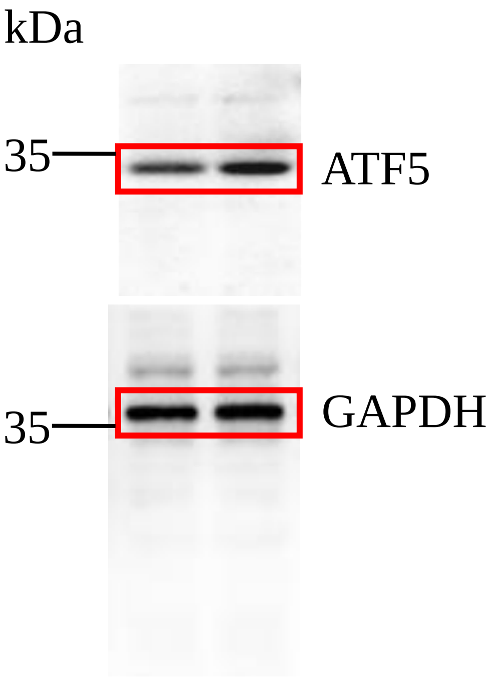

Figure 6 e

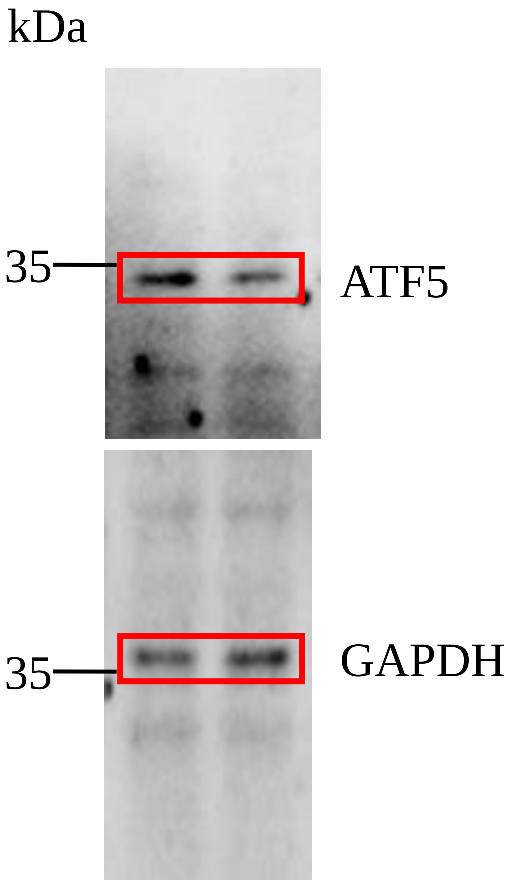

Figure 7 c

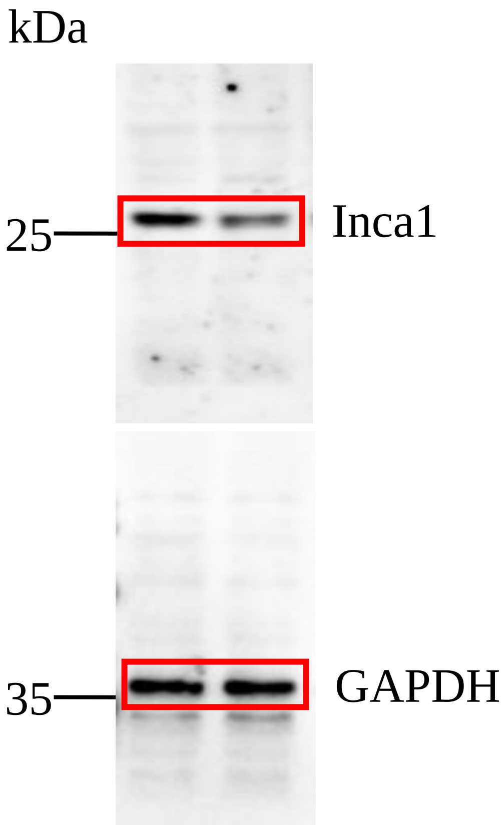

Figure 7 f

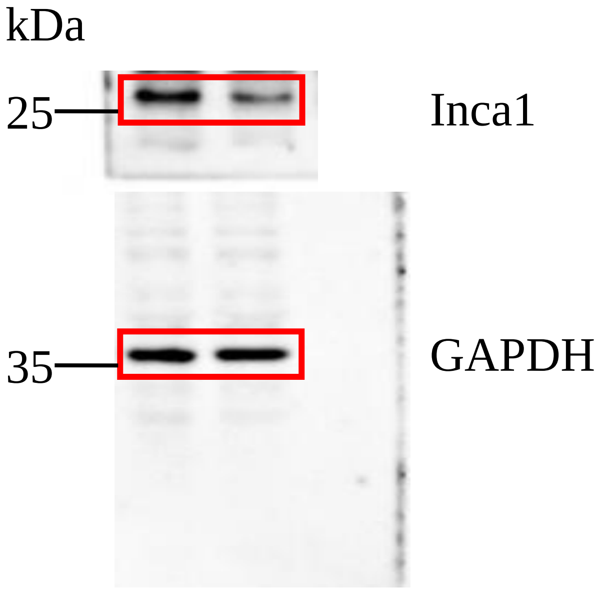

Figure 7 g

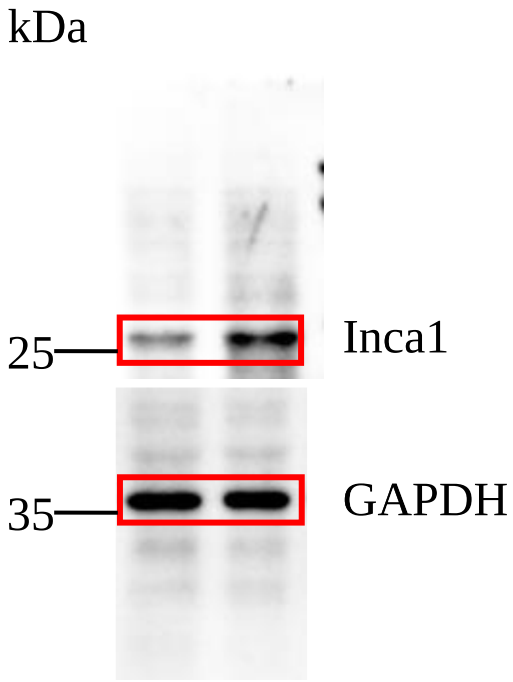

Figure 7 h

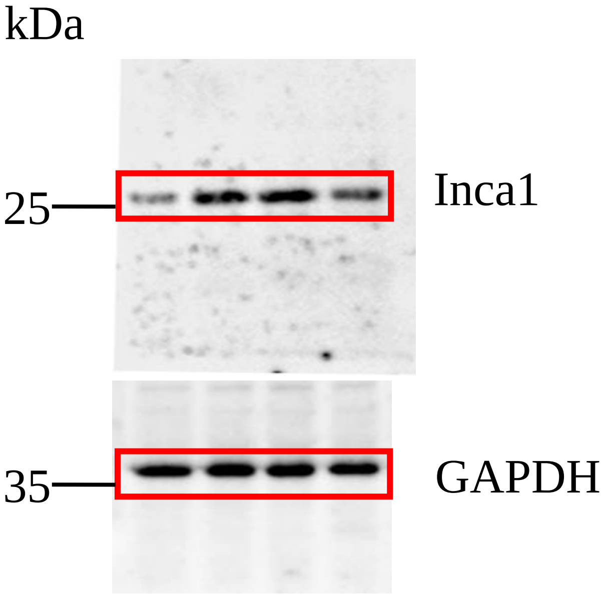

Supplementary Fig. 1 b

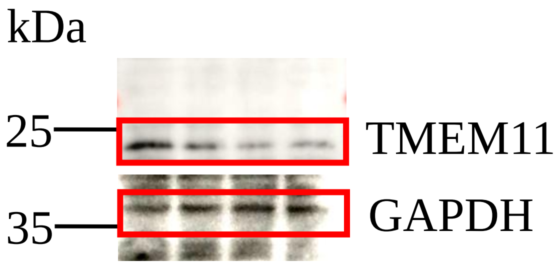

Supplementary Fig. 1 c

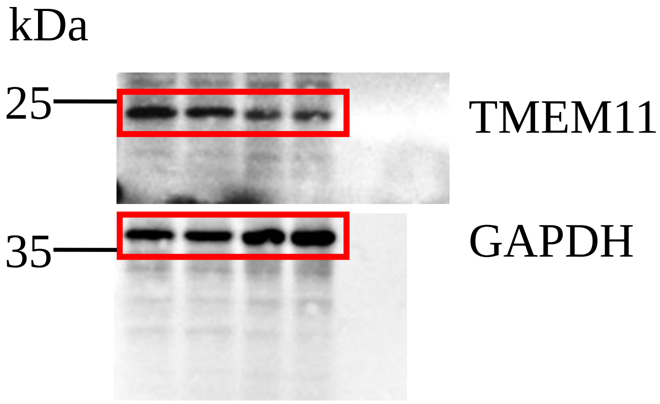

Supplementary Fig. 2 d

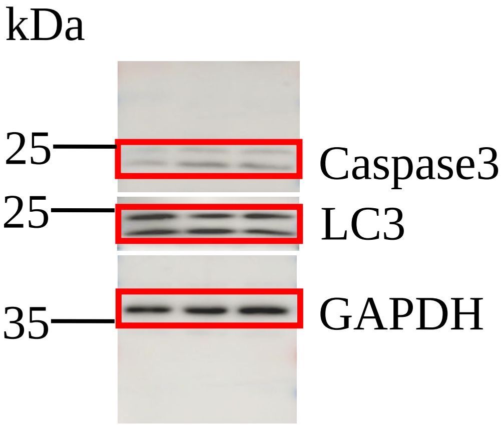

Supplementary Fig. 4 d

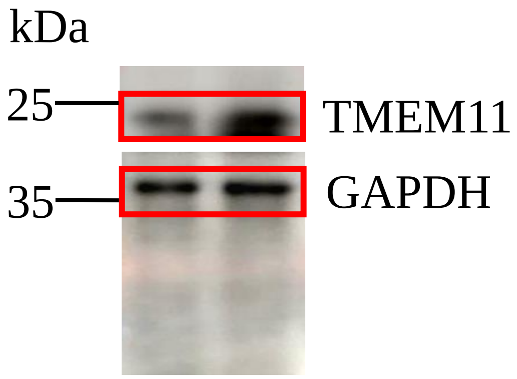

Supplementary Fig. 4 e

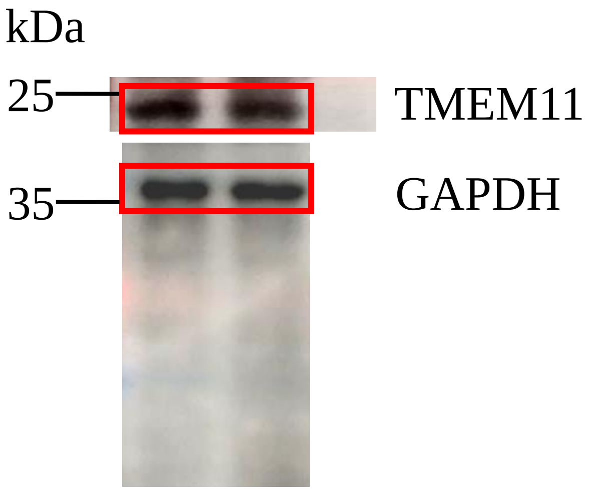

Supplementary Fig. 5 a

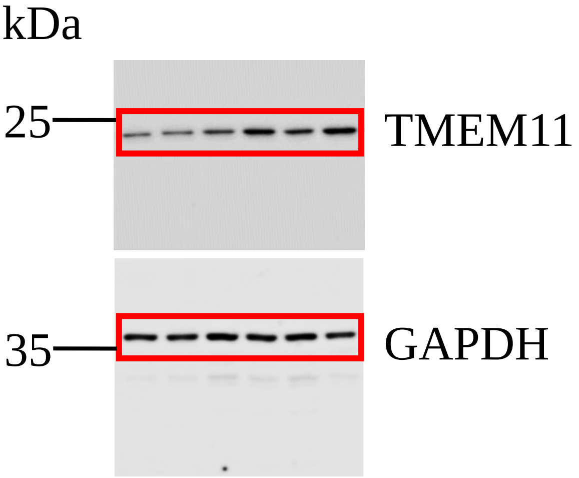

Supplementary Fig. 7 a

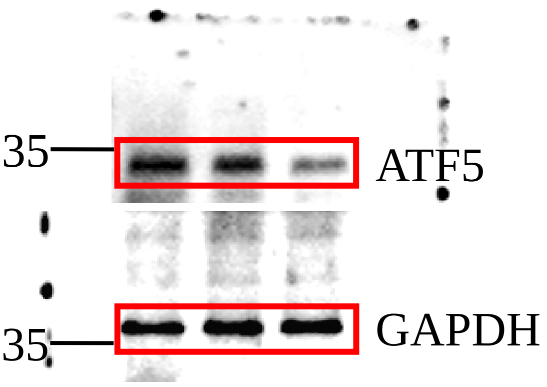

Supplementary Fig. 7 c

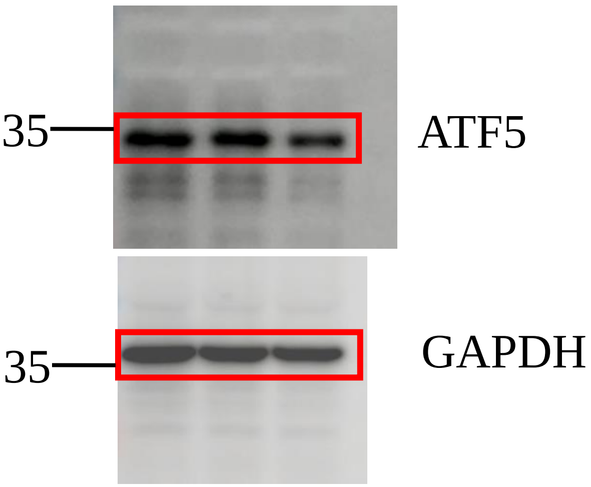

Supplementary Fig. 7 f

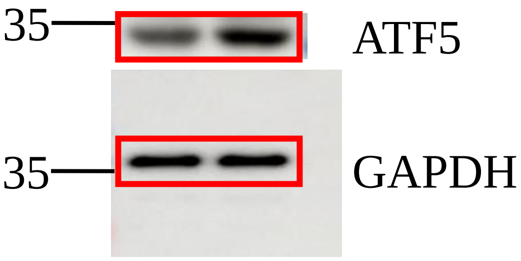

Supplementary Fig. 7 g

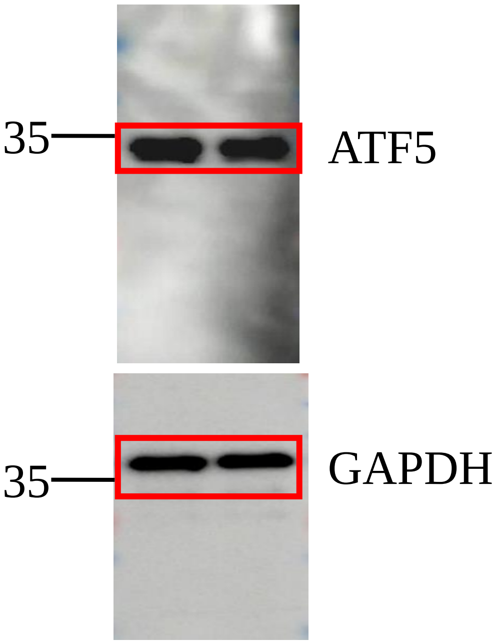

Supplementary Fig. 7 i

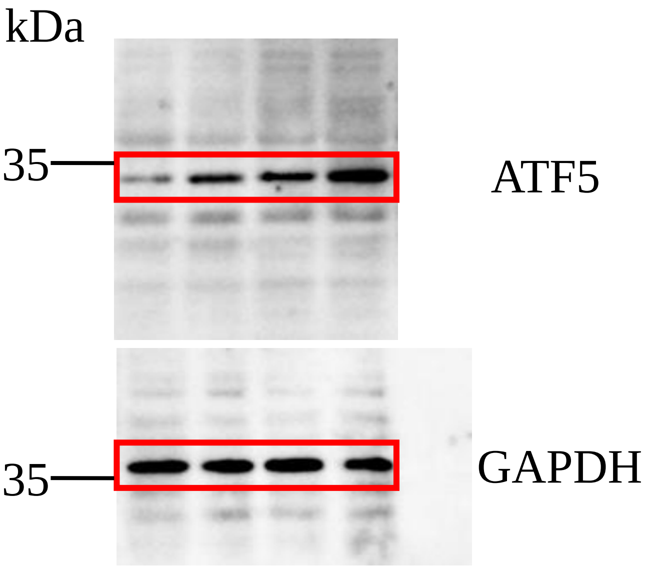

Supplementary Fig. 8 d

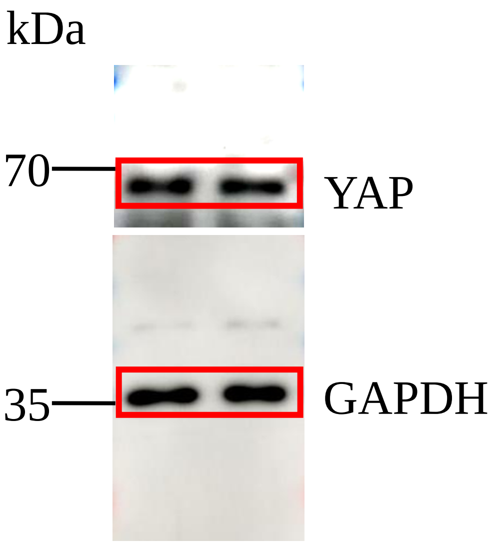

Supplementary Fig. 8 e

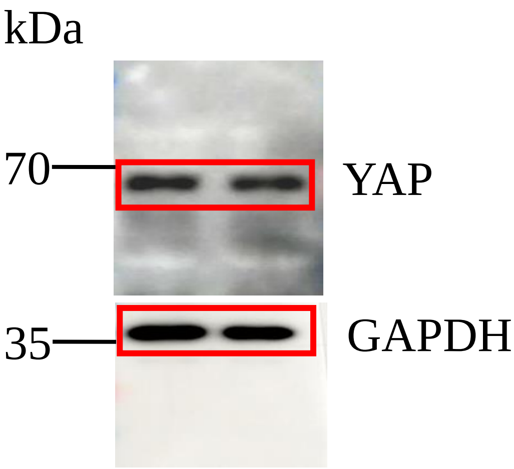

Supplementary Fig. 8 f

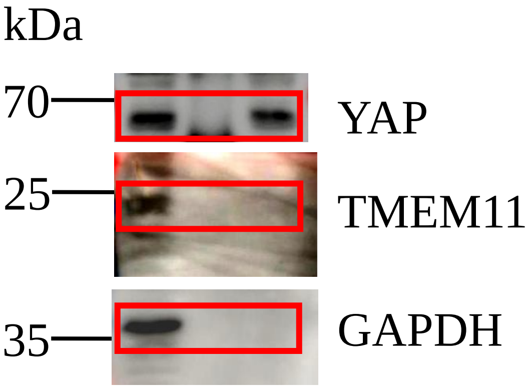

Supplementary Fig. 9 d

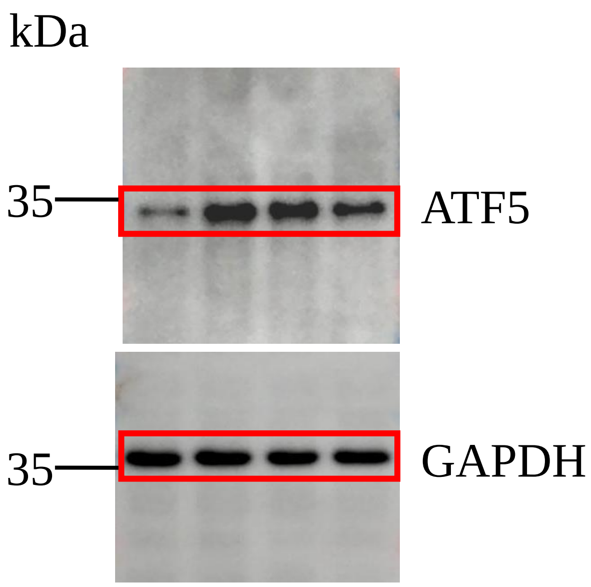

Supplement: Supplementary file 15 — Original Data File [file 41418_2023_1179_MOESM15_ESM.pdf]
